# Curcumin Alleviates DSS-Induced Anxiety-Like Behaviors via the Microbial-Brain-Gut Axis

**DOI:** 10.1155/2022/6244757

**Published:** 2022-03-18

**Authors:** Fan Zhang, Yanlin Zhou, Haitao Chen, Hao Jiang, Feini Zhou, Bin Lv, Maosheng Xu

**Affiliations:** ^1^The First School of Clinical Medicine of Zhejiang Chinese Medical University, Hangzhou, Zhejiang 310053, China; ^2^Department of Radiology, The First Affiliated Hospital of Zhejiang Chinese Medical University (Zhejiang Provincial Hospital of Traditional Chinese Medicine), Hangzhou, Zhejiang 310003, China; ^3^Key Laboratory of Digestive Pathophysiology of Zhejiang Province, The First Affiliated Hospital of Zhejiang Chinese Medical University, Hangzhou, Zhejiang 310006, China; ^4^Department of Gastroenterology, The First Affiliated Hospital of Zhejiang Chinese Medical University (Zhejiang Provincial Hospital of Traditional Chinese Medicine), Hangzhou, Zhejiang 310003, China; ^5^The Cancer Hospital of the University of Chinese Academy of Sciences (Zhejiang Cancer Hospital), Hangzhou, Zhejiang 310022, China; ^6^Institute of Basic Medicine and Cancer (IBMC), Chinese Academy of Sciences, Hangzhou, Zhejiang 310022, China

## Abstract

The anxiety and depression caused by inflammatory bowel diseases (IBD) are known to greatly affect the mental health of patients. The mechanism of psychiatric disorders caused by IBD is not fully understood. Previous research has suggested that the gut microbiome plays a key role in IBD. Curcumin is a yellow polyphenol extracted from the rhizome of the ginger plant, which has been shown to have effects against both depression and anxiety. Research has indicated that curcumin affects the gut microbiome and exerts antianxiety and neuroprotective effects through the microbiota-gut-brain axis (MGB). However, whether curcumin can alleviate the psychiatric disorders caused by IBD and how curcumin affects the MGB axis through the gut microbiota have not been fully understood. Therefore, this study was aimed at determining the metabolic parameters and microbiological environment in the peripheral and central nervous system to determine the effects of curcumin against anxiety induced by dextran sulfate sodium salt (DSS) in mice. To elaborate on the link between the gut microbiota and how curcumin alleviates anxiety-like behaviors, we performed a fecal microbiota transplantation (FMT) experiment. The results suggested that curcumin can effectively relieve anxiety-like behaviors caused by DSS in mice. Further, curcumin treatment can alleviate disturbances in the gut microbiota and systemic disorders of lipid metabolism caused by DSS. Finally, through FMT, we verified that curcumin increased phosphatidylcholine in the prefrontal cortex of the mice and alleviated DSS-induced anxiety-like behaviors by modulating specific gut microbiota. We also revealed that *Muribaculaceae* may be a key part of the gut microbiota for curcumin to alleviate DSS-induced anxiety-like behaviors through the MGB axis.

## 1. Introduction

Inflammatory bowel diseases (IBD) describe a variety of conditions including Crohn's disease (CD) and ulcerative colitis (UC). Generally, these conditions are immunological in basis and present with a waxing-waning nature of symptoms and intestinal inflammation. The prevalence of clinically relevant anxiety and depressive symptoms has been noted to be higher in those with IBD, and up to 30% of patients with IBD suffer from psychiatric disorders such as anxiety and depression [1]. These psychiatric disorders were associated with more aggressive IBD [2], nonobedience to medical therapy [3], and an increased risk of suicide [4]. By contrast, psychiatric symptoms caused by IBD tend to receive less attention than gastrointestinal symptoms. Although the etiology of IBD and psychiatric symptoms in IBD patients are not fully defined, several studies suggest that interactions among the microbiota-gut-brain (MGB) axis likely affect the development of IBD [5] and psychiatric symptoms in IBD patients [6]. The gut microbiota is thought to be a virtual organ of the body [7]. In human beings, 99% of the genes are microbes [8, 9], the gut microbiota can not only affect the function of the digestive system, and evidence has also suggested that the gut microbiota can modulate brain function and host behaviors [10, 11]. Therefore, some scholars suggest the MGB axis, which involves bidirectional communication between the microbiota and brain [12].

Curcumin (1,7-bis(4-hydroxy-3-methoxyphenyl)-1,6-heptadiene-3,5-dione) is the main natural polyphenol extracted from the rhizome of Curcuma longa and other Curcuma spp. [13]. Curcuma longa has been traditionally used as a medicinal herb due to its antioxidant [14] and anti-inflammatory properties [15]. Currently, curcumin is most commonly used as a dietary spice in addition to an additive in cosmetic and pharmaceutic products [16]. In addition, curcumin is also believed to hold medicinal properties against many diseases, including gastrointestinal [17], cardiovascular [18], and mental diseases [19]. However, the bioavailability of curcumin is unstable, and the concentration of curcumin in plasma is very low after oral administration of large doses of curcumin [20]. High concentrations of curcumin have been detected in the gastrointestinal tract after oral administration [21]. Supplementation of curcumin significantly enriched beneficial bacteria such as Butyricicoccus, a butyrate producing genus in the intestinal tract [22], and reduced Ruminococcus, and Mucispirillum, which were implicated in the development of obesity and diabetes [23, 24]. These findings were the basis to suggest that curcumin directly affects the gut microbiome despite its low systemic bioavailability.

While curcumin can relieve inflammation and digestive tract symptoms caused by IBD [25, 26], whether curcumin can alleviate IBD-induced anxiety-like behaviors has not been reported. Hence, we used dextran sulfate sodium salt (DSS) to induce acute colitis in mice. Then, 16S rDNA gene sequencing was used to identify acute changes in the microbiome. Metabolic studies were also used to determine the role of curcumin on the MGB axis in DSS-treated mice by systematic analysis of feces, serum, and prefrontal cortex (PFC). Furthermore, to clarify whether curcumin can alleviate IBD-induced anxiety symptoms by regulating gut microbiota, fecal microbiome transplantation (FMT) was carried out.

## 2. Materials and Methods

### 2.1. Animal Treatment

**Figure 1:**
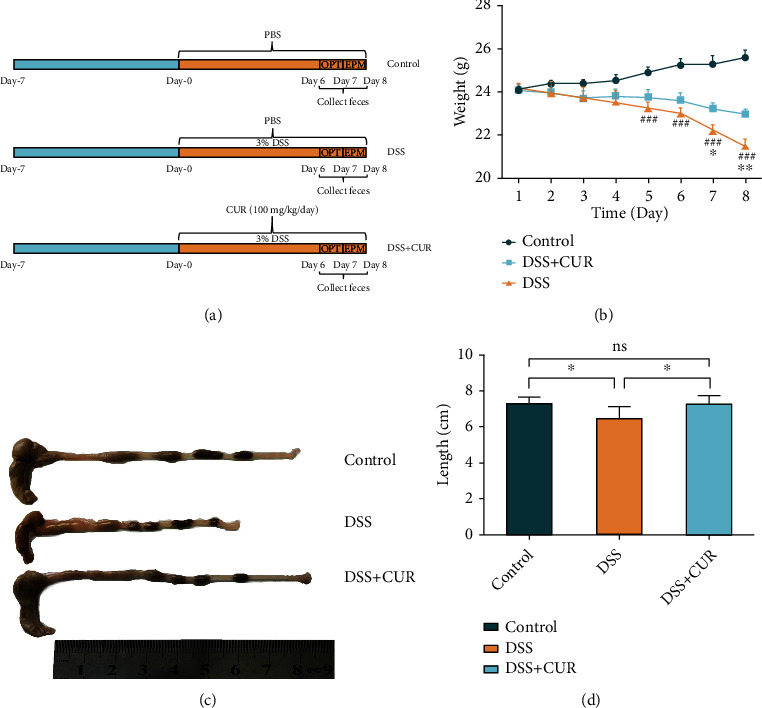
The drug treatment schedule and basic conditions of mice after drug intervention. (a) Design of curcumin experiment to DSS-treated mice (*n* = 10/group). (b) Body weight of control group, DSS group, and DSS + CUR group (DSS vs. control: ^#^*P* < 0.05; ^##^*P* < 0.01; ^###^*P* < 0.001. DSS + CUR vs. DSS: ^∗^*P* < 0.05, ^∗∗^*P* < 0.01). (c, d) The colon length of the control group, DSS group, and DSS + CUR group (^∗^*P* < 0.05; ^∗∗^*P* < 0.01; ^∗∗∗^*P* < 0.001). Data were represented as mean ± SEM.

C57BL/6 mice (*n* = 30) were randomly divided into 3 groups with 10 mice each: (1) control group; (2) DSS group; (3) DSS + CUR group. The drinking water of DSS group and DSS + CUR group was supplemented with 3% DSS to induce acute colitis for 8 days. The DSS + CUR group was administered 100 mg/Kg/day curcumin (Sigma M5250) dissolved with phosphate-buffered saline for 8 days from the beginning of DSS administration until the day to the last DSS treatment. A volume of 100 *μ*L/10 g body weight was administered, while the control and DSS group mice received the same amount of phosphate buffered saline. Fresh fecal samples were collected on the 6th to 8th day. The body weights were recorded once daily. On the 6th and 7th day of DSS administration, behavioral tests were performed. All mice were anesthetized by an intraperitoneal injection of 1% pentobarbital sodium (50 mg/kg) and sacrificed by the cervical dislocation method on the 8th day after initiation of DSS. Serum and brain tissues were collected, and all samples were stored at -80°C until further use. The drug treatment schedule is shown in Figure 1(a). This experiment was supervised and approved by the Experimental Animal Ethical Committee of the Zhejiang Chinese Medical University (approved no.: ZSLL-2018-014).

### 2.2. Open Field Test (OFT)

The OFT was designed to assess the level of anxiety in mice by using an open environment model. Each mouse was randomly selected and individually tested in an open-field apparatus (50 × 50 cm^2^ and 40 cm in height). The bottom of the apparatus was equally divided into 16 squares. Each subject was put in a corner of the apparatus for 1 minute to acclimate. All spontaneous activities were recorded (5 min) and calculated by an automated motor activity recording tracker (SMART 3.0, Panlab, Barcelona, Spain). Considering the total distance of movement as an indicator of locomotor activity, the proportion of the time and distance spent in the center of the square (25% of the inner surface area) was used as an indicator of anxiety-like behaviors. The apparatus was cleaned with 75% ethanol solution before and after each test.

### 2.3. Elevated Plus-Maze (EPM) Test

The EPM apparatus consists of a “+” shaped maze elevated 50 cm above the floor with two oppositely positioned open arms, two oppositely positioned closed arms, and a 5 cm × 5 cm center area. Each arm was 30 cm long and 5 cm wide. The closed arms had 15 cm high walls while the open arms had 3 mm high ledges. Each mouse was placed in the center of the apparatus, facing an open arm. All spontaneous activities were recorded (5 min) and calculated by SMART 3.0, as previously described. The frequency of open arm entries and time spent in the open arms was used as an index of anxiety-like behavior. The apparatus was cleaned with 75% ethanol solution before and after each test.

### 2.4. 16S rRNA Miseq Sequencing and Bioinformatic Analysis

The total genomic DNA of the gut microbiome was extracted from 200 mg of fecal samples using the E.Z.N.A. ®Stool DNA Kit (D4015, Omega, Inc., USA) following the manufacturer's instructions. Then, the V3-V4 region of the bacterial 16S rRNA gene in each sample was amplified with primers 341F (5′-CCTACGGGNGGCWGCAG-3′) and 805R (5′-GACTACHVGGGTATCTAATCC-3′). Subsequently, a mixture of PCR products was purified by AMPure XT beads (Beckman Coulter Genomics, Danvers, MA, USA) and quantified by Qubit (Invitrogen, USA). The 16S rRNA gene sequences were analyzed using Illumina NovaSeq platform. According to the unique bar code and truncated by cutting off the barcode and primer sequence of the samples, the paired-end reads were assigned to the samples. Paired-end reads were merged using Fast Length Adjustment of SHort reads (FLASH). According to fqtrim (version 0.94), the raw reads data were quality filtered under certain filtering conditions to obtain a high-quality, clean label. Then, the chimeric sequences were filtered by Vsearch software (version 2.3.4). These sequences were clustered to operational taxonomic units (OTUs) with a similarity over 97% assigned to the same OTUs. Alpha diversity and beta diversity were calculated by QIIME2, during which the same sequences were extracted randomly through reducing the number of sequences to the minimum of some samples, and the relative abundance (X bacteria count/total count) was used in bacteria taxonomy. Alpha diversity and beta diversity were analyzed by QIIME2 process. The sequence alignment of species annotation was performed by Blast, and the alignment database was SILVA and NT-16S.

### 2.5. PFC and Serum Liquid Chromatography Mass Spectrometry (LC-MS) Analysis and Data Processing

The metabolites were extracted from the prefrontal cortex (PFC) and serum with 50%. Briefly, 20 *μ*L of sample was precooled by 120 *μ*L 50% methanol extraction, vortexed for 1 min, and incubated at 24°C for 10 min; the supernatants were extracted after centrifugation at 4,000 g for 20 min and transferred into new 96-well plates for the liquid chromatography mass spectrometry (LC-MS) analysis to identify the metabolites. Chromatographic separations were performed with an ultraperformance liquid chromatography (UPLC) system (SCIEX, UK). Phase separation was reversed by an ACQUITY UPLC T3 column (100 mm∗2.1 mm, 1.8 *μ*m, Waters, UK). Metabolites eluted from the chromatographic column were detected by a high-resolution tandem mass spectrometer, Triple TOF 5600 plus (SCIEX, UK). The Q-TOF was operated in both positive and negative ion modes. The TOF mass range was from 60 to 1200 Da. Additionally, in order to evaluate the stability of the LC-MS during the whole acquisition, a quality control sample (pool of all samples) for every 10 samples was acquired. The KEGG (https://www.kegg.jp/) and Human Metabolome Database (http://www.hmdb.ca/, HMDB) were used to annotate the metabolites using the exact molecular mass data (*m*/*z*) of samples. *T* tests were conducted to detect differences in metabolite concentrations between 2 phenotypes. The *P* value was adjusted for multiple tests using an FDR (Benjamini-Hochberg). Supervised PLS-DA was conducted through metaX to discriminate the different variables between groups. The VIP value was calculated. A VIP cut-off value of 1.0 was used to select important features.

### 2.6. Feces ^1^H NMR Spectroscopy Analysis and Data Processing

The preparation of fecal metabolomics samples was referred to in previous literature. Briefly, 100 mg thawed fecal material from each mouse was mixed with 0.8 mL phosphate buffer saline (PBS) containing 10% deuterated water (D2O 99.8% with 0.05 mM/100 mL sodium 3-trimethylsilylpropionate-d4 as chemical shift reference; SIGMA). The mixture was kept on the ice for 15 min and then dissolved for 10 cycles (one cycle includes 20 s ultrasound, 10 s crash, and 30 s rest). Then, the suspension solution of feces was centrifuged at 13000 RPM for 10 min at 4°C. The supernatant was extracted for further testing.

All ^1^H NMR spectra were recorded by Bruker 600 MHz AVANCE III spectrometer equipped with a 5 mm BBFO probe at 25°C. Shimming and proton pulse calibration were performed automatically for each sample before data acquisition. ^1^H NMR spectra were received using NOESYPR 1D pulse sequence with water suppression. The data was processed using Bruker Topspin 3.2.

Free induction decays (FIDs) from ^1^H NMR of the feces were multiplied by a 0.3 Hz exponential line broadening prior to Fourier transformation. All obtained NMR spectra were manually phased, baseline corrected, and referenced to TSP (*δ* = 0.0) within MestReNova 12 (Mestrelab Research SL, Spain). The integral region of the spectrum was set between 0.0 and 9.0 ppm, with a spectral region of 4.5-5.0 ppm to eliminate the effects of imperfect water suppression. Due to the deviation of metabolite concentration in the feces of each mouse, each bucket was internally normalized to the total sum of the spectral integrals prior to pattern recognition analysis. The characteristic peaks of all fecal metabolites were determined based on related literature [27, 28] and HMDB.

### 2.7. Pseudogerm-Free Mice Modeling

20 mice (8 weeks) were randomly assigned into two groups (*n* = 10): FMT (DSS) group and FMT (DSS + CUR). Both groups of mice were treated with normal water mixed with antibiotics (1 g/L metronidazole, 0.2 g/L ciprofloxacin, 1 g/L neomycin, and 0.5 g/L vancomycin) for 14 consecutive days. All antibiotics were purchased from Meilun Bio (Dalian, China), and drinking water was renewed every 2 days. After 14 days of antibiotic treatment, the antibiotics containing water were replaced with normal water and the pseudogerm-free mice were transplanted with donor microbiota.

### 2.8. Gut Microbiota Transplantation

200 mg of the fecal samples were suspended in 3 mL of PBS. The mixture was mixed and centrifugated at 1000g for 10 min at 4°C. The FMT (DSS) group mice were administered with the fecal suspension from DSS group, and the FMT (DSS + CUR) group mice were administered with the fecal suspension from DSS + CUR group. The microbiota supernatants were transplanted into each mouse by gavage with 0.2 mL/mice for 5 days.

### 2.9. OFT and EPM Test

The experimental methods were the same as the study 2.1 and 2.2.

### 2.10. Lipidomics Analysis and Data Analysis

The PFC collected samples were thawed on ice, and lipids were extracted with isopropanol (IPA) buffer. Briefly, 20 *μ*L of the sample was extracted with 120 *μ*L of precooled IPA, vortexed for 1 min, and incubated at room temperature for 10 min; the extraction mixture was then stored overnight at -20°C. After centrifugation at 4,000 g for 20 min, the supernatants were transferred into new 96-well plates and diluted to 1 : 10 with IPA/acetonitrile (ACN)/H2O (2 : 1 : 1, *v* : *v* : *v*). In addition, pooled PFC samples were also prepared by combining 10 *μ*L of each extraction mixture. All samples were acquired by the LC-MS system-followed machine orders. Firstly, all chromatographic separations were performed using an ultraperformance liquid chromatography (UPLC) system (SCIEX, UK). A Kinetex UPLC C18 column (100 mm∗2.1 mm, 100 A, Phenomenex, UK) was used for the reversed phase separation. The column oven was maintained at 55°C. The flow rate was 0.3 mL/min, and the mobile phase consisted of solvent A (ACN : water = 6 : 4 + 0.1%formic acid) and solvent B (IPA : ACN = 9 : 1 + 0.1%formic acid). The injection volume for each sample was 2 *μ*l. A high-resolution tandem mass spectrometer TripleTOF 5600plus (SCIEX, UK) was used to detect metabolites eluted from the column. The Q-TOF was operated in both positive and negative ion modes. The TOF mass range was from 100 to 2000 Da. Furthermore, in order to evaluate the stability of the LC-MS during the whole acquisition, a quality control sample (pool of all samples) for every 10 samples was acquired. *T* tests were conducted to detect differences in metabolite concentrations between 2 phenotypes. The *P* value was adjusted for multiple tests using an FDR (Benjamini-Hochberg). Supervised PLS-DA was conducted through metaX to discriminate the different variables between groups. The VIP value was calculated. A VIP cut-off value of 1.0 was used to select important features.

### 2.11. Statistical Analysis

The experimental data were processed and analyzed by GraphPad Prism 6 software (Version 6.01). All values were expressed as mean ± SEM. Data of multiple groups such as weight, length of the colon, and behaviors were assessed by one-way analysis of variance (ANOVA). A value of *P* < 0.05 was considered as statistically significant. 16S rRNA and metabolomics data between two groups were analyzed by unpaired Student's *t*-test. PCA and PLS-DA analyses were performed using SIMCA software (Version 14.1). The differential metabolites were filtered by variable influence on projection (VIP) selection according to the PLS-DA, and only metabolites with VIP > 1.0 and *P* < 0.05 were identified as differential metabolites. Further, R language (R version 3.5.2) was used to make the classified tree heatmap. Enrichment analysis of KEGG metabolic pathways were performed using metaboAnalyst (https://www.metaboanalyst.ca/home.xhtml). Venn diagrams were drawn using Bioinformatics (http://bioinformatics.psb.ugent.be/webtools/Venn/). Pearson correlation analysis results were generated using IBM SPSS Statistics 25.0 software. The correlation network diagram was drawn by R language (R version 3.5.2).

## 3. Results

### 3.1. Curcumin Suppresses DSS-Induced Anxiety-Like Behaviors

**Figure 2:**
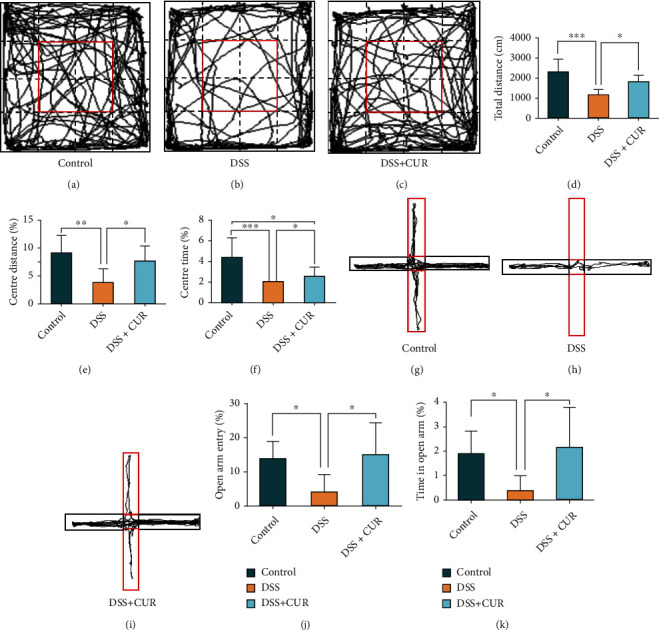
OFT and EPM tests after mice were treated with DSS and curcumin. (a–c) Motion tracks of the control group, DSS group, and DSS + CUR group in the OFT. (d–f) Results of OFT, respectively, were total distance, center distance (%), and center time (%). (g–i) Motion tracks of control group, DSS group, and DSS + CUR group in the EPM test. (j, k) Results of EPM test, respectively, were open arm entry (%) and time in the open arm (%). Data were represented as mean ± SEM. ^∗^*P* < 0.05; ^∗∗^*P* < 0.01; ^∗∗∗^*P* < 0.001.

The body weight of DSS and DSS + CUR mice was statistically significantly lower after 8 days of DSS free drinking water intervention. On the 7th and 8th day, the body weight of mice in the DSS + CUR group was significantly different compared to those in the DSS group (Figure 1(b)). In addition, the colonic length of DSS group was also significantly decreased compared to the control group. However, curcumin treatment significantly reversed the effect of DSS on colonic length (Figures 1(c) and 1(d)). Additionally, the OFT test showed that DSS-treated mice significantly decreased total distance, center distance (%), and center time (%). Moreover, the EPM test revealed that DSS-treated mice also decreased open arm entry (%) and time in the open arm (%). However, curcumin treatment significantly reversed the DSS effects on the anxiety-like behaviors measured by OFT and EPM test (Figures 2(a)–2(k)).

### 3.2. Curcumin Reprogramed the Gut Microbiota in DSS Treatment Mice

#### 3.2.1. Alpha Diversity

**Figure 3:**
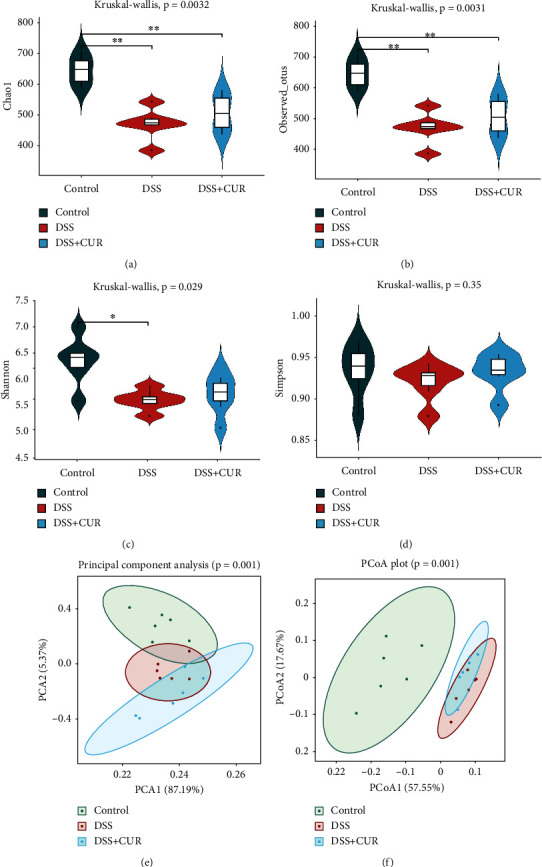
The alpha diversity and beta diversity of gut microbiota in three groups (*n* = 6/group). (a–d) Alpha diversity of Chao1, Observed_otus, Shannon diversity, and Simpson evenness. (e, f) Beta diversity of PCA and PCoA. ^∗^*P* < 0.05, ^∗∗^*P* < 0.01.

Alpha diversity analysis included the Observed species, Chao1, Shannon, and Simpson indices; the Observed species and Chao1 were used to represent the richness of the bacterial community, while Shannon and Simpson were used to describe the diversity of the bacterial community. In our study, alpha diversity, as measured by the Observed species, Chao1, and Shannon indices, was significantly reduced after treatment with DSS. While treatment with curcumin increased alpha diversity indexes, there was no statistical difference compared with the DSS group (Figures 3(a)–3(d)). These data suggest that DSS treatment decreased the richness and diversity of gut microbiota, but curcumin treatment did not increase them.

#### 3.2.2. Beta Diversity

The beta diversity was determined to examine the consistency of the microbial community between different samples. Based on the unweighted UniFrac principal components analysis (PCA) and principal coordinates analysis (PCoA), control, DSS, and DSS + CUR groups presented a distinct clustering of microbiota community structure (Figures 3(e) and 3(f)). These results suggest that the gut microbiota structure was changed after DSS and curcumin intervention.

#### 3.2.3. Microbial Community Composition

**Figure 4:**
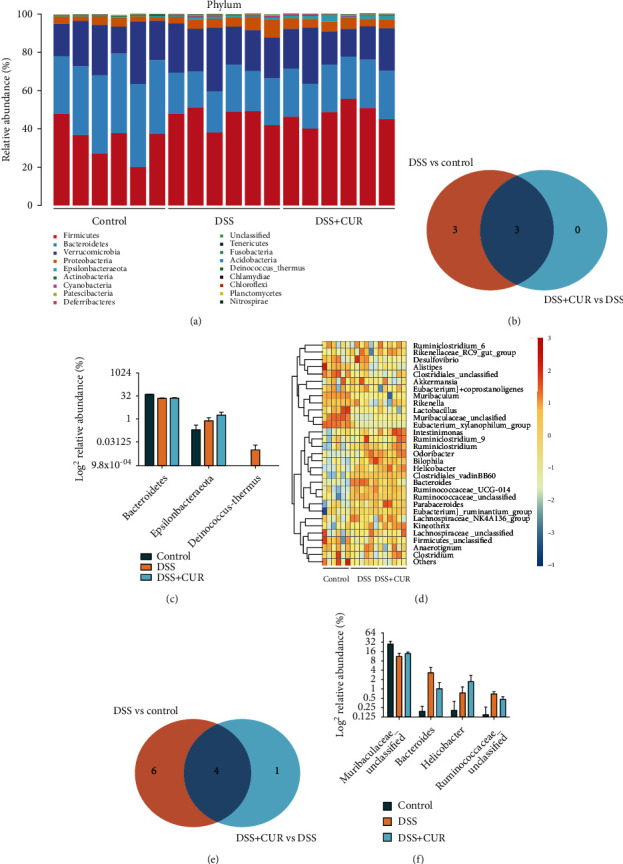
Composition of the gut microbiota among groups (*n* = 6/group). (a) Bacterial taxonomic profiling at the phylum level of three groups. (b) Venn diagrams showed the number of differential gut microbiota at the phylum level between the DSS vs. the control group (orange) and DSS + CUR vs. DSS group (light blue) and their shared differential gut microbiota (navy blue). (c) Relative abundances of differential gut microbiota at phylum level between DSS vs. the control group and DSS + CUR vs. DSS group. (d) Heatmap of the relative abundances of various bacterial genera identified in three groups. The red or blue colors represent the high relative or low relative of abundance in each sample. (e) Venn diagrams showed the number of differential gut microbiota at genera level between the DSS vs. the control group (orange) and DSS + CUR vs. the DSS group (light blue) and their shared differential gut microbiota (navy blue). (f) Relative abundances of differential gut microbiota at genera level between DSS vs. control group and DSS + CUR vs. DSS group.

Figure 4(a) showed the microbial community composition of each sample at the phylum level. Compared with the control group, DSS treatment decreased the relative abundance of *Bacteroidetes* and *Actinobacteria*, increased the relative abundance of *Firmicutes*, *Cyanobacteria*, *Epsilonbacteraeota*, and *Deinococcus-Thermus*. In contrast, curcumin treatment corrected the relative abundance of *Bacteroidetes and Deinococcus-Thermus* changed by DSS (Figures 4(b) and 4(c)).

Using the observed differences between the three groups at the phylum level, the abundance of the top 30 genera was depicted using a heatmap (Figure 4(d)). The Venn analysis of Figure 4(e) demonstrated the regulation of 4 differential gut microbiota between the DSS vs. control group and DSS + CUR vs. DSS group. Notably, at the genus level, DSS reduced *Muribaculaceae_unclassified* and increased the amount of *Bacteroides* and *Ruminococcaceae_unclassified*. Meanwhile, curcumin treatment could partly reverse these changes (Figure 4(f)).

**Figure 5:**
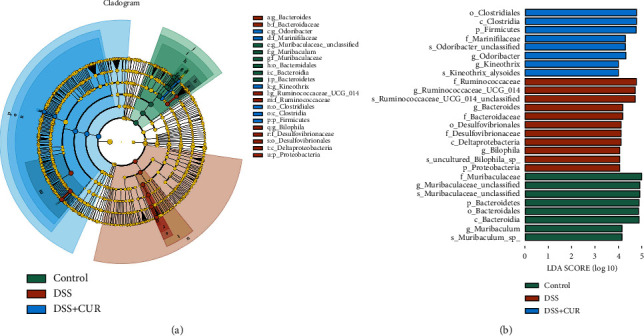
The LEfSe analysis of microbial abundance among three groups (*n* = 6/group). (a) A cladogram generated by LEfSe analysis; (b) taxa with a significant difference between the three groups were detected by LEfSe analysis with a LDA threshold score of 4 and a significant *P* < 0.05.

The Linear Discriminant Analysis Effect Size (LEfSe) algorithm was performed to identify the characteristic bacteria which were specific for each group. As shown in Figures 5(a) and 5(b), a total of 21 prokaryotic clades were selected with an LDA threshold greater than 4 and *P* < 0.05. Taxa with significantly higher abundance in the control group all belonged to Phylum *Bacteroidetes* including genus *Muribaculum* and *Muribaculaceae_unclassified*. DSS group showed a high abundance of Phylum *Proteobacteria* and genera *Bilophila*, genera *Ruminococcaceae_UCG_014*, and genera *Bacteroides*. DSS + CUR group showed a high abundance of Phylum *Firmicutes* and genera *Kineothrix*, and genera *Odoribacter*.

### 3.3. Curcumin Changed Fecal Metabolites in DSS Mice

Fecal metabolites and their respective characteristics were determined in accordance with prior studies and the HMDB database. All in all, 39 fecal metabolites were detected. The PCA scatter plot (Supplement Figure 1A) suggested differences between the control group and the DSS and DSS + CUR groups. In addition, the degree of separation and cluster characteristics between the three groups were shown using the PLS-DA score plot (Supplement Figure 1B-1D), indicating that the fecal metabolites among these three groups showed significant differences. The two PLS-DA models were determined by the parameters R2Y and Q2Y, in which both models R2Y and Q2Y equate to 1 indicating effective models (Supplement Figure 1E-1F). Significantly differential metabolites were determined according to the following criteria: PLS − DA VIP (variable importance in the projection) > 1, *P* < 0.05. With the criteria mentioned above, 18 and 14 metabolites were highlighted between the DSS vs. control group and the DSS + CUR vs. DSS group (Supplement data 1 and 2), respectively. Venn analysis revealed 8 metabolites as key differential metabolites specific to curcumin treatment (Supplement Figure 1G). These key differential metabolites were shown in Supplement Figure 1H.

### 3.4. PFC Metabolomic Changes in DSS and DSS + CUR Mice

**Figure 6:**
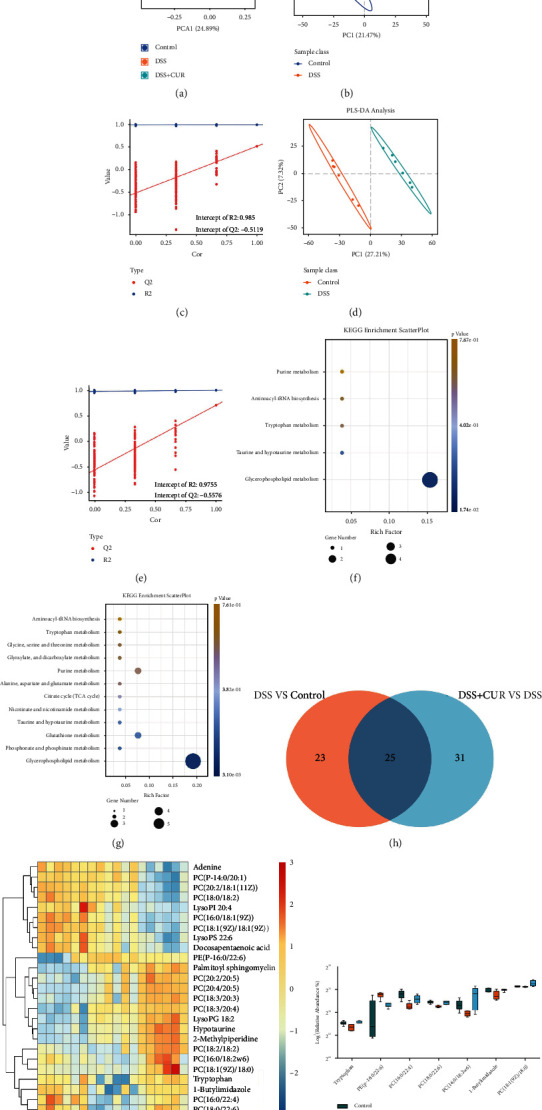
PFC metabolic analysis (*n* = 6/group). (a) The PCA scatter plot of PFC samples among the control, DSS. and DSS + CUR group. (b, c) The PLS-DA score plots and validation plot of PFC samples obtained from the DSS vs. control group. (d, e) The PLS-DA score plots and validation plot of PFC samples obtained from DSS + CUR vs. DSS group. (f) PFC KEGG enrichment scatter plot of DSS vs. control group. (g) PFC KEGG enrichment scatter plot of DSS + CUR vs. DSS group. (h) Venn diagrams showed the differential metabolites. Differential metabolites were identified based on *P* < 0.05 and VIP > 1 as the filter of DSS vs. control group and DSS + CUR vs. DSS group. (i) Heatmap of the relative abundances of differential metabolites among three groups. The red or blue color represents the high relative or low relative of abundance in each sample. (j) The change of the key PFC metabolites for the efficacy of curcumin.

The metabolomic analysis yielded 4037 characteristics in the positive ion mode (PIM) and 4595 characteristics in the negative ion mode (NIM). The metabolic parameters were utilized to build a batch query against the human metabolome database (HMDB) in the two-stage (MS2) mass spectrometry analysis and annotated 275 MS2 features in PIM and 265 MS2 features in NIM. In order to understand the differences between the 3 groups, the metabolomic data were submitted to the SIMCA-P software package for PCA and PLS-DA analysis. PCA reveled distinct factors between all three groups (Figure 6(a)). According to the PLS-DA model, DSS group and control group, DSS + curcumin, and DSS groups all clearly separated in the *x*-axis direction. The quality of the two PLS-DA models was determined by the parameters R2Y and Q2Y, in which both models R2Y and Q2Y equated to 1 indicating the effectiveness of the model (Figure 6(b)–6(e)).

Metabolites which differed significantly were determined in accordance with the following criteria: PLS − DA VIP > 1, *P* < 0.05. We identified 48 metabolites that were differentially expressed in the DSS group as compared with the control group. Among these metabolites, 21 metabolites (Trimethadione, Hypotaurine, trans-Muconic acid, and so forth) were upregulated, while the other 27 metabolites were downregulated (Supplementary data 3). As shown in Figure 6(f), KEGG pathway analysis revealed 1 significantly enriched pathway based on the above 48 metabolites, with “glycerophospholipid metabolism” being the most significantly enriched pathway. Further, we identified 56 metabolites that were differentially expressed in the DSS + CUR group as compared with the DSS group. Among these metabolites, 32 metabolites (phosphatidylcholine (18 : 0/22 : 6), phosphatidylcholine (16 : 0/22 : 4(7Z,10Z,13Z,16Z)), palmitoyl sphingomyelin, and so forth) were upregulated, while other 24 metabolites were downregulated (Supplementary data 4). Based on the above 32 metabolites, KEGG pathway analysis revealed 1 significantly enriched pathway, with “glycerophospholipid metabolism” being the most significantly enriched pathway (Figure 6(g)). The Venn analysis of Figure 6(h) demonstrates the regulation of 25 differential metabolites between the DSS vs. control group and DSS + CUR vs. DSS group. Additionally, heatmaps showed the concentrations of these 25 differential metabolites in three groups (Figure 6(i)). Among them, 7 metabolites (PE(P-16 : 0/22 : 6), PC(16 : 0/22 : 4), PC(18 : 0/22 : 6), PC(16 : 0/18:2w6), 1-butylimidazole, tryptophan, and PC(18 : 1(9Z)/18 : 0)) showed an opposite trend to DSS after curcumin treatment, and therefore, these 5 metabolites were identified as the key metabolites that specific to curcumin treatment (Figure 6(j)).

### 3.5. Serum Metabolomic Changes in DSS and DSS + CUR Mice

The metabolomic analysis yielded 4037 characteristics in the positive ion mode (PIM) and 4595 characteristics in the negative ion mode (NIM). The characteristics of the metabolomic analysis were used as the batch query against the human metabolome database (HMDB) in the two-stage (MS2) mass spectrometry analysis and annotated 275 MS2 features in PIM and 273 MS2 features in NIM. In order to understand the differences between the control group, DSS group, and DSS + curcumin groups, the metabolomic data were submitted to the SIMCA-P software package for PCA and PLS-DA analysis. PCA analysis demonstrated a significant difference between the control and DSS groups, but no significant difference between the DSS group and the curcumin group (Supplement Figure 2A). According to the PLS-DA model, DSS group and the control group, DSS + curcumin, and DSS groups all separated in the *x*-axis direction. The two PLS-DA models were determined by parameters R2Y and Q2Y, in which both models R2Y and Q2Y equated to 1 indicating effective models (Supplement Figure 2B-2E).

Metabolites that differed significantly were characterized by the following criteria: PLS − DA VIP > 1, *P* < 0.05. We identified 34 metabolites that were differentially expressed in DSS group as compared with the control group (Supplementary data 5). As shown in Supplement Figure 2F, the KEGG pathway analysis revealed 2 significantly enriched pathways based on the above 34 metabolites, respectively, the purine metabolism and glycerophospholipid metabolism pathways. Further, we identified 30 metabolites that were differentially expressed in DSS + CUR group as compared with the DSS group (Supplementary data 6). Based on the above 30 metabolites, KEGG pathway analysis revealed 2 significantly enriched pathways, with “glycerophospholipid metabolism” being the most significantly enriched pathway (Supplement Figure 2G). The Venn analysis of Supplement Figure 2H demonstrates the regulation of 10 differential metabolites between the DSS vs. control group and DSS + CUR vs. DSS group. Among these 10 different metabolites, 5 metabolites (proline, LysoPC 16 : 1, dexpanthenol, 3-hydroxydodecanoic acid, and 1-mercapto-2-propanone) showed an opposite trend to DSS after curcumin treatment, and therefore, these 5 metabolites were identified as the key metabolites that were specific to curcumin treatment (Supplement Figure 2I).

### 3.6. Fecal Transplanted from DSS + CUR Mice Showed Lower Anxiety-Like Behaviors Than That of Transplanted from DSS Mice

**Figure 7:**
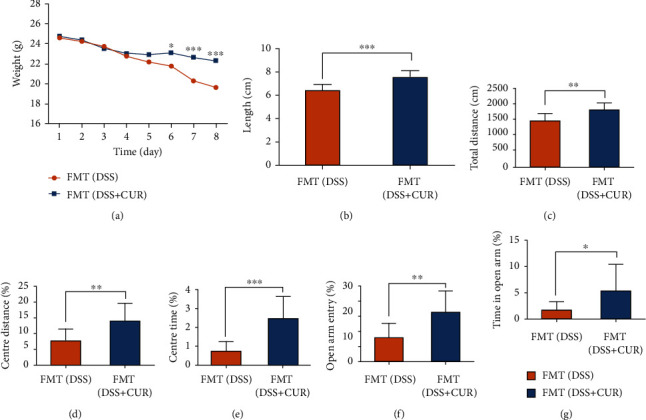
Basic conditions and behavioral tests after FMT. (a) Body weight of FMT (DSS) and FMT (DSS + CUR) group. (b) The colon length of FMT (DSS) and FMT (DSS + CUR) group. (c–e) Results of OFT, respectively, were total distance, center distance (%), and center time (%). (f, g) Results of EPM test, respectively, were open arm entry (%) and time in the open arm (%). Data were represented as mean ± SEM. ^∗^*P* < 0.05; ^∗∗^*P* < 0.01; ^∗∗∗^*P* < 0.001.

As shown in Figure 7(a), on the 6th, 7th, and 8th day, the body weight of FMT (DSS + CUR) group was statistically higher than FMT (DSS) group. In addition, the colonic length of FMT (DSS + CUR) group was also longer compared to the FMT (DSS) group (Figure 7(b)). Moreover, FMT (DSS + CUR) group significantly outperformed FMT (DSS) group in both OFT and EPM test (Figures 7(c)–7(g)). The data above suggested that curcumin alleviated the anxiety-like behaviors caused by DSS regulating the gut microbiota.

### 3.7. FMT Effectively Reshaped Gut Microbiota of Pseudogerm-Free Mice

As shown in the Supplement Figure 3A-3D, the alpha diversity of gut microbiota in FMT (DSS + CUR) group was significantly higher than that in the FMT (DSS) group. The beta diversity of the gut microbiota was also significantly different between the two groups after FMT (Supplement Figures 3E, 3F).

The difference of the microbial community composition of the phylum level between FMT (DSS + CUR) group and FMT (DSS) group was shown in Supplement Figure 4A. At the phylum level, the content of *Firmicutes*, *Actinobacteria*, and *Cyanobacteria* in the FMT (DSS + CUR) group were significantly higher than that in FMT (DSS) group, while the content of *Verrucomicrobia* was significantly lower (Supplement Figures 4B, 4C). The difference in the microbial community between the FMT (DSS + CUR) group and FMT (DSS) group was shown in Supplement Figure 4D. Among these gut microbiota, 14 genera (*Muribaculaceae_unclassified*, *Bilophila*, *Bacteroides*, and so forth) in FMT (DSS + CUR) group were upregulated compared to FMT (DSS) group, while other 5 genera (*Akkermansia*, *Parabacteroides*, *Escherichia-Shigella*, *Parasutterella*, and *Blautia*) were downregulated (Supplement Figures 4E, 4F). These results indicated that the gut microbiota of DSS + CUR and DSS group were successfully colonized in the intestinal tracts of pseudosterile mice. The content of *Muribaculaceae* was significantly increased after feeding curcumin and after feeding feces from DSS + CUR group; this suggests that *Muribaculaceae* may be the key microbiota for curcumin to alleviate DSS-induced anxiety-like behaviors.

### 3.8. Fecal Transplanted from DSS + CUR Mice Significantly Improved PFC Lipid Metabolism

**Figure 8:**
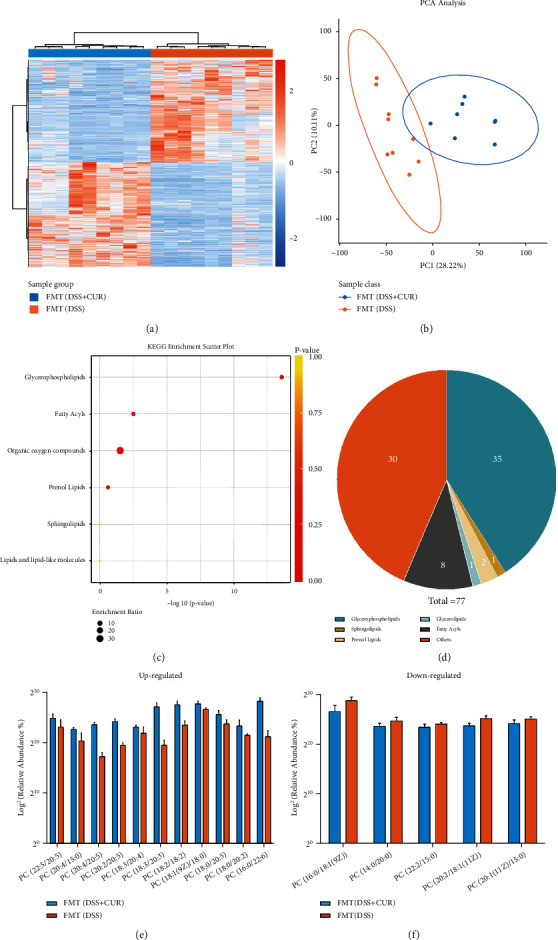
PFC lipid metabolism analysis (*n* = 9/group). (a) The heatmap of the differences in the PFC metabolic profile between FMT (DSS) group and FMT (DSS + CUR) group in PIM and NIM. (b) The PCA scatter plot of PFC samples between FMT (DSS) group and FMT (DSS + CUR) group. (c) KEGG enrichment scatter plot of FMT (DSS + CUR) vs. FMT (DSS) group. (d) The pie chart showed the categorization of differential metabolites. (e, f) The change of differential metabolites belonging to the PC species of FMT (DSS + CUR) vs. FMT (DSS) group.

The lipid metabolomic analysis yielded 11484 characteristics in the PIM and NIM groups. Meanwhile, a difference between the two aforementioned groups was also observed using the heatmap (Figure 8(a)). Moreover, PCA analysis shows that lipid metabolic signatures of PFC were significantly separated in the *x*-axis direction (Figure 8(b)). All in all, 77 metabolites differentially expressed metabolites (VIP > 1.0, *P* < 0.05) between two groups were identified (Supplementary data 7). KEGG pathway analysis revealed 4 significantly enriched pathways, with “glycerophospholipid metabolism” being the most significantly enriched pathway (Figure 8(c)). Among these metabolites, 35 metabolites were glycerophospholipids, 8 were fatty acyls, 2 were prenol lipids, 1 was a glycerolipid, and 1 was a sphingolipids (Figure 8(d)). Through the first part of the experiment, we found that curcumin mainly regulates the phosphatidylcholine (PC) content in PFC of DSS group mice. Therefore, after FMT, we focused on the altered content of PC in the PFC lipid metabolomics. As shown in Figures 8(e) and 8(f), the differential lipid metabolites consisted of 16 PC-species metabolites; among them, 11 PC-species metabolites were increased, and 5 PC-species metabolites were decreased in the FMT (DSS + CUR) group compared with the FMT (DSS) group.

## 4. Discussion

In recent decades, the gut microbiota and neuroscience have become more and more closely linked. Although the MGB axis is a relatively new concept, it has been suggested that the environment in the gut microbiome has a significant impact on the behavior of the subject. Here, we observed that DSS-treated mice presented with altered lipid metabolism profiles along with microbial compositions. In particular, the PC content in PFC plays an important role in DSS-induced anxiety-like behaviors. Through two parts of the experiment, we found that curcumin could alleviate anxiety-like behaviors in DSS-treated mice by regulating the gut microbiota, which provided a strong basis for the hypothesis that curcumin acts directly on the gut microbiota. Moreover, curcumin treatment ameliorated “glycerophospholipid metabolism” of the MGB axis, and most importantly, PC content deficits induced by DSS, which may underlie its antianxiety effects.

Consistent with prior literature, a greater prevalence of gut microbiome dysregulation in DSS-induced IBD model mice was also seen. Specifically, the contents of *Firmicutes*, *Cyanobacteria*, *Epsilonbacteraeota*, and *Deinococcus-Thermus* increased, while the contents of *Bacteroidetes* and *Actinobacteria* content significantly dropped. The etiology of IBD is not clear; numerous studies have shown that the gut microbiota plays an important role in the development and progression of IBD [29, 30]. Although it has been recognized that DSS can significantly alter the diversity, richness, and composition of gut microbiota, the specific impact on the composition of the microflora is still controversial. The effect of DSS on the two most abundant groups of bacteria at the phylum level, *Firmicutes* and *Bacteroidetes*, was highly controversial, and many experimental results in the literature were contradictory. For example, Chen et al. [31] observed that DSS could reduce the abundance of *Bacteroidetes*, but had no significant effect on *Firmicutes*. However, Munyaka et al. [32] found that the level of DSS was able to lower the level of *Bacteroidetes* while increasing *Firmicutes*. DSS could decrease the abundance of *Bacteroidetes* and increase the abundance of *Firmicutes*, while Dou et al. [33] found that DSS could increase the abundance of *Bacteroidetes*. Park et al. [34] concluded that the alteration of the composition of the gut microbiota by different concentrations of DSS was highly variable. This likely explains why this experimental data differed from previous studies. However, the effect of DSS on gut microbiota is recognized and significant, which was similar to the alteration of gut microbiota in clinical IBD patients [35].

From the two parts of the microbial community composition and LEfSe analysis, we found that the *Muribaculaceae* family was significantly higher in abundance in the control group mice, and curcumin treatment can effectively reverse the decrease of *Muribaculaceae* abundance caused by DSS. Other studies indicated that taking curcumin orally can increase the level of helpful bacteria including *Lactobacilli*, *Bifidobacteria*, and butyrate-producing bacteria [36]. In addition, the richness and diversity of gut microbiota were increased alongside a reduction in pathogenic bacteria associated with a variety of systemic diseases, such as Enterobacteria, Prevotellaceae, and Rikenellaceae [37–39]. However, for the first time, we discovered that curcumin can achieve therapeutic effects by increasing the abundance of *Muribaculaceae*. Bacteria within the *Muribaculaceae* family also designated as S24-7 or Candidatus Homeothermaceae are one of the dominant bacterial taxa in the mice gut microbial composition [40]. Studies have shown that increasing the abundance of *Muribaculaceae* can improve the intestinal microenvironment, increase the output of metabolites beneficial to the intestinal tract, and prolong the life span of mice [41]. To clarify the function of the *Muribaculaceae* family, Ormerod et al. [42] found that the genome of the *Muribaculaceae* contains genes related to the fermentation pathway; data from the gene library also suggest that *Muribaculaceae* can produce succinic acid, acetic acid, and propionic acid in the intestine. *Muribaculaceae* also have the protective ability of antioxidation and antistress properties [42]. A decline of the abundance of *Muribaculaceae* has also been associated with a number of diseases. Numerous clinical studies have shown that the abundance of *Muribaculaceae* is reduced in a variety of inflammatory diseases such as IBD [43] and type I diabetes [44]. Moreover, these findings were also observed in mice models of IBD induced by DSS [45], although we have proved that *Muribaculaceae* was the key bacteria for curcumin to alleviate anxiety-like behaviors in the IBD mice. However, *Muribaculaceae*, as an anaerobic bacterium, is difficult culture and grow in the intestinal tract of mice. Therefore, it remains to be further investigated how curcumin produces its therapeutic effect through *Muribaculaceae*.

To clarify how curcumin alleviated anxiety-like behaviors in IBD mice via the MGB axis, we integrated pathway enrichment data from the metabolomics of the PFC and serum to elucidate the key pathways. These two metabolic pathways were all enriched into the glycerophospholipids metabolism. The brain is a lipid-rich organ, in which lipids account for approximately half of the brain dry weight and play crucial roles in maintaining several complex and key physiological functions [46], such as transmitting nerve impulses [47], involving in the myelin sheath formation [47], and acting as the signaling molecules [48]. The lipids of the CNS are mainly composed of glycerophospholipids, glycerolipids, sphingolipids, sterols, and fatty acids [47], which glycerophospholipids rank amongst the most abundant lipids in neuron cells and serve as both structural and signaling molecules of the membrane [49]. Disturbances in lipid function can be a trigger for a range of conditions [50]. Previous clinical and animal experiments have found that stress and psychiatric disorders may also be caused by changes in lipid metabolism [51, 52]. IBD is characterized by dysregulation of the gut microbiota [30], which can regulate lipid metabolism and absorption at the intestinal mucosal level [53]. Additionally, the gut microbiota can change the lipid metabolism of the liver by regulating the expression of genes related to lipid metabolism, and the short-chain fatty acids produced by gut microbiota are also the substrates of liver lipid production [54]. Due to the lack of a gut microbiome, germ-free mice showed higher levels of anxiety-like behaviors and significantly different lipid metabolism in the PFC from normal mice [55]. We observed similar findings in DSS-induced IBD mice as in GF mice. However, after treatment with curcumin, the content of short-chain fatty acids in the feces of the DSS group significantly increased. In addition, the condition of lipid metabolism in the prefrontal lobes and serum of DSS mice was also significantly improved. Additionally, through the FMT experiments and the analysis of lipid metabolomic data between mice receiving microbiota from DSS mice and DSS + curcumin mice group, the glycerophospholipids metabolism was also the most significantly enriched metabolic pathway. Therefore, we have reason to believe that curcumin may improve systemic lipid metabolism by regulating the gut microbiota and alleviating anxiety-like behaviors.

Our fecal metabolomics analysis suggested that DSS treatment significantly reduced the choline content in feces. In animals, choline is an essential nutrient. Most of the choline is exogenous through diet [56], while small amounts of choline can also be endogenously produced through the phosphatidylethanolamine N-methyltransferase (PEMT) pathway. Choline is essential for maintaining the health of rodents and humans, and choline deficiency can cause muscle damage [57], nonalcoholic fatty liver disease [58], and mental disorders [59]. In addition, when fed a choline-deficient diet to mice that lack of PEMT, mice developed steatosis and steatohepatitis in just one day, and died of liver failure three days later [60]. However, a decrease in fecal choline content does not indicate that the body is in choline deficiency, and our serum and PFC metabolomics data also did not show a decrease in choline levels. But the metabolomics data of the PFC provided favorable evidence for choline deficiency. That is, the PC content in the PFC of mice was significantly lower in the DSS group when compared with the control. Most of the choline from food intake and endogenous production is converted into PC, which accounts for 95% of the total choline pool in most animal tissues [61]. When choline content is insufficient, the CNS choline is preferentially used for the synthesis of acetylcholine, which is used in cholinergic neurotransmission, and that process may restrict the production of phosphatidylcholine [62]. In addition, the body converts PC to choline through the PEMT/phospholipase reactions, thus compensating for choline content [63]. PC is the highest content of the phospholipids, which mainly concentrates in the myelin and neuronal membranes [49]. PC is involved in the composition of cellular lipid bilayers, and the PC content is also important for maintaining the stability of neuronal cell membranes and for maintaining the activity of ion channels and receptors [64]. Decreased PC levels have been associated with a variety of psychiatric and neurological disorders. For example, decreased PC content in the brain has been observed in Parkinson's disease [65], Alzheimer's disease [66], and stroke [67]. A chronic stress study showed that the PFC is the brain region most affected by chronic stress, and that rats showed a significant decrease in PC levels in the PFC along with anxiety-like behaviors [68]. This is similar to what we observed in the PFC of mice treated with DSS. Moreover, PFC PC levels were significantly increased after curcumin treatment, and the same phenomenon was observed after the pseudosterile mice received DSS + CUR mice feces. Hence, these findings provided a strong rationale for the efficacy of curcumin through the MGB axis, and decreased prefrontal PC content may be a marker for the development of anxiety-like behaviors in DSS-treated mice.

In conclusion, our results suggest that curcumin can effectively relieve anxiety-like behaviors caused by DSS in mice. Curcumin treatment can alleviate disturbances in the gut microbiota and systemic disorders of lipid metabolism caused by DSS. The FMT experiment verified that curcumin can increase the PC content in PFC and alleviate DSS-induced anxiety-like behaviors by modulating specific gut microbiota. We also revealed that *Muribaculaceae* may be a key component of the gut microbiota for curcumin to alleviate DSS-induced anxiety-like behaviors through the MGB axis. In addition, PFC PC content was highly negatively associated with DSS-induced anxiety-like behaviors. Future work should aim to determine whether prefrontal PC is a biomarker of psychiatric disorders caused by IBD.

## Data Availability

The data used to support the findings of this study are included within the article and supplementary information files. Additional data used to support the findings of this study are available from the corresponding author upon request.

## Supplementary Materials

Supplementary 1 Supplementary File 1 Tables: (a) comparison of fecal metabolites between DSS group and control group. (b) Comparison of fecal metabolites between DSS + CUR group and DSS group. (c) Differential PFC metabolites between DSS and control groups. (d) Differential PFC metabolites between DSS + CUR and DSS groups. (e) Differential serum metabolites between DSS and control groups. (f) Differential serum metabolites between DSS + CUR and DSS groups. (g) Differential PFC lipid metabolites between FMT (DSS) and FMT (DSS + CUR) groups.

Supplementary 2 Figure supplement 1: fecal metabolic analysis between control, DSS, and DSS + CUR groups (*n* = 8/group).

Supplementary 3 Figure supplementary 2: serum metabolic analysis between control, DSS, and DSS + CUR groups (*n* = 6/group).

Supplementary 4 Figure supplementary 3: the alpha diversity and beta diversity of gut microbiota between FMT (DSS) group and FMT (DSS + CUR) group (*n* = 6/group).

Supplementary 5 Figure supplementary 4: composition of gut microbiota between FMT (DSS) group and FMT (DSS + CUR) group (*n* = 6/group).
