# Supplementary material for: Curcumin Alleviates DSS-Induced Anxiety-Like Behaviors via the Microbial-Brain-Gut Axis
Source: Oxid Med Cell Longev. 2022 Mar 18;2022:6244757. doi: 10.1155/2022/6244757 (PMC8957039; doi:10.1155/2022/6244757)
Supplement: Supplementary 1 — Supplementary File 1 Tables: (a) comparison of fecal metabolites between DSS group and control group. (b) Comparison of fecal metabolites between DSS + CUR group and DSS group. (c) Differential PFC metabolites between DSS and control groups. (d) Differential PFC metabolites between DSS + CUR and DSS groups. (e) Differential serum metabolites between DSS and control groups. (f) Differential serum metabolites between DSS + CUR and DSS groups. (g) Differential PFC lipid metabolites between FMT (DSS) and FMT (DSS + CUR) groups. [file 6244757.f1.doc]

**Table (a). Feces Metabolism Between Control and DSS Group**

| **Metabolites** | **Differential metabolites** | **VIP** | **P value** | **Control**  **Mean** | **DSS**  **Mean** | **SEM of difference** |
| --- | --- | --- | --- | --- | --- | --- |
| β-glucose |  | 0.612 | 0.562 | 2.123 | 1.286 | 1.408 |
| Xanthine |  | 0.204 | 0.773 | 0.473 | 0.423 | 0.170 |
| Valine | * | 1.038 | 0.007 | 4.359 | 1.918 | 0.780 |
| Valerate | * | 1.083 | 0.006 | 5.550 | 2.278 | 0.999 |
| Uracil | * | 1.300 | 0.000 | 1.686 | 0.435 | 0.222 |
| Tyrosine |  | 0.596 | 0.144 | 2.138 | 1.624 | 0.332 |
| Tryptophan | * | 1.138 | 0.002 | 2.753 | 0.864 | 0.479 |
| Trimethylamine |  | 1.128 | 0.150 | 0.964 | 1.299 | 0.220 |
| Taurine |  | 0.866 | 0.049 | 0.803 | 0.390 | 0.192 |
| Succinate | * | 1.407 | 0.000 | 5.970 | 0.504 | 0.743 |
| Propionate | * | 1.282 | 0.000 | 33.419 | 13.734 | 4.077 |
| Proline | * | 1.200 | 0.001 | 7.308 | 2.259 | 1.128 |
| Phenylalanine |  | 0.664 | 0.326 | 2.083 | 1.271 | 0.797 |
| Methylamine | * | 1.380 | 0.000 | 0.360 | 0.106 | 0.037 |
| Methionine | * | 1.036 | 0.006 | 1.184 | 0.628 | 0.171 |
| Methanol |  | 0.806 | 0.171 | 0.765 | 0.935 | 0.118 |
| Lysine | * | 1.233 | 0.000 | 5.780 | 1.658 | 0.882 |
| Leucine |  | 0.770 | 0.218 | 5.130 | 3.460 | 1.295 |
| Lactose |  | 0.766 | 0.054 | 9.855 | 4.566 | 2.515 |
| Lactate |  | 0.927 | 0.005 | 18.331 | 9.970 | 2.484 |
| Isoleucine |  | 0.564 | 0.200 | 0.914 | 0.563 | 0.261 |
| Isobutyrate | * | 1.196 | 0.000 | 3.805 | 1.844 | 0.385 |
| Hypoxanthine |  | 0.976 | 0.010 | 1.713 | 0.640 | 0.358 |
| Glycine | * | 1.093 | 0.003 | 1.430 | 0.349 | 0.300 |
| Glutamine |  | 0.968 | 0.014 | 2.281 | 0.924 | 0.483 |
| Glucose |  | 0.904 | 0.051 | 3.556 | 0.855 | 1.263 |
| Fumarate |  | 0.297 | 0.803 | 0.131 | 0.140 | 0.034 |
| Formate |  | 0.798 | 0.059 | 0.654 | 0.200 | 0.221 |
| Ethanol |  | 0.434 | 0.318 | 1.979 | 1.285 | 0.669 |
| D-Xylose | * | 1.299 | 0.000 | 0.888 | 0.428 | 0.084 |
| D-galactose |  | 0.264 | 0.715 | 0.284 | 0.353 | 0.185 |
| Choline | * | 1.603 | 0.000 | 4.320 | 1.791 | 0.218 |
| Butyrate | * | 1.271 | 0.000 | 39.399 | 11.239 | 5.437 |
| Aspartate |  | 0.694 | 0.383 | 2.014 | 0.833 | 1.311 |
| Arabinose |  | 0.930 | 0.080 | 0.388 | 1.171 | 0.415 |
| Alanine | * | 1.090 | 0.005 | 5.988 | 3.161 | 0.844 |
| Acetate | * | 1.183 | 0.001 | 99.930 | 61.544 | 8.818 |
| 3-hydroxyphenylacetate |  | 0.720 | 0.319 | 0.900 | 0.381 | 0.503 |
| 2-oxoisovalerate | * | 1.153 | 0.001 | 0.489 | 0.220 | 0.066 |

**Table (b). Feces Metabolism Between DSS + CUR and DSS Group**

| **M**etabolites | **Differential metabolites** | **VIP** | **P value** | **DSS + CUR**  **Mean** | **DSS**  **Mean** | **SEM of difference** |
| --- | --- | --- | --- | --- | --- | --- |
| β-glucose |  | 1.063 | 0.059 | 10.191 | 1.286 | 4.340 |
| Xanthine | * | 1.043 | 0.048 | 0.753 | 0.423 | 0.152 |
| Valine | * | 1.223 | 0.016 | 3.729 | 1.918 | 0.664 |
| Valerate |  | 0.917 | 0.223 | 3.151 | 2.278 | 0.685 |
| Uracil |  | 0.768 | 0.158 | 0.688 | 0.435 | 0.169 |
| Tyrosine |  | 0.934 | 0.235 | 2.041 | 1.624 | 0.336 |
| Tryptophan | * | 1.124 | 0.032 | 1.824 | 0.864 | 0.402 |
| Trimethylamine |  | 0.833 | 0.150 | 0.949 | 1.299 | 0.230 |
| Taurine |  | 1.126 | 0.259 | 0.656 | 0.390 | 0.226 |
| Succinate | * | 1.229 | 0.018 | 1.836 | 0.504 | 0.495 |
| Propionate |  | 0.079 | 0.913 | 13.526 | 13.734 | 1.863 |
| Proline |  | 1.166 | 0.471 | 2.720 | 2.259 | 0.623 |
| Phenylalanine |  | 0.703 | 0.212 | 2.020 | 1.271 | 0.572 |
| Methylamine |  | 1.010 | 0.262 | 0.163 | 0.106 | 0.048 |
| Methionine |  | 0.794 | 0.481 | 0.736 | 0.628 | 0.150 |
| Methanol |  | 0.442 | 0.945 | 0.941 | 0.935 | 0.089 |
| Lysine |  | 0.851 | 0.123 | 2.800 | 1.658 | 0.696 |
| Leucine | * | 1.221 | 0.025 | 6.398 | 3.460 | 1.173 |
| Lactose |  | 0.755 | 0.198 | 10.775 | 4.566 | 4.593 |
| Lactate |  | 0.991 | 0.677 | 10.816 | 9.970 | 1.987 |
| Isoleucine |  | 0.769 | 0.210 | 0.918 | 0.563 | 0.270 |
| Isobutyrate | * | 1.186 | 0.002 | 2.801 | 1.844 | 0.256 |
| Hypoxanthine |  | 0.525 | 0.861 | 0.676 | 0.640 | 0.203 |
| Glycine | * | 1.364 | 0.018 | 1.023 | 0.349 | 0.252 |
| Glutamine |  | 1.316 | 0.599 | 1.113 | 0.924 | 0.351 |
| Glucose | * | 1.061 | 0.044 | 2.128 | 0.855 | 0.574 |
| Fumarate | * | 1.102 | 0.034 | 0.333 | 0.140 | 0.082 |
| Formate | * | 1.288 | 0.011 | 0.758 | 0.200 | 0.191 |
| Ethanol |  | 1.215 | 0.094 | 2.323 | 1.285 | 0.578 |
| D-Xylose |  | 0.789 | 0.942 | 0.433 | 0.428 | 0.068 |
| D-galactose |  | 0.193 | 0.769 | 0.420 | 0.353 | 0.225 |
| Choline | * | 1.332 | 0.012 | 2.381 | 1.791 | 0.203 |
| Butyrate | * | 1.188 | 0.020 | 17.125 | 11.239 | 2.250 |
| Aspartate |  | 0.736 | 0.263 | 1.475 | 0.833 | 0.551 |
| Arabinose | * | 1.239 | 0.022 | 6.883 | 1.171 | 2.211 |
| Alanine | * | 1.514 | 0.001 | 5.265 | 3.161 | 0.528 |
| Acetate |  | 0.599 | 0.288 | 53.334 | 61.544 | 7.433 |
| 3-hydroxyphenylacetate |  | 0.687 | 0.709 | 0.338 | 0.381 | 0.115 |
| 2-oxoisovalerate |  | 0.631 | 0.269 | 0.316 | 0.220 | 0.084 |

**Table (c). Differentially PFC Metabolites Between Control and DSS Group**

| **MS2Metabolite** | **p value** | **VIP** | **Control Mean** | **DSS Mean** | **SEM of difference** | **Trends after DSS treatment** |
| --- | --- | --- | --- | --- | --- | --- |
| Tryptophan | 0.0193 | 1.0676 | 221970.0 | 122185.0 | 33859.1 | ↓ |
| Trimethadione | 0.0440 | 1.0044 | 1870096.0 | 3000227.0 | 417993.0 | ↑ |
| Trans-Muconic acid | 0.0473 | 4.3375 | 147990.0 | 2117504.0 | 650248.0 | ↑ |
| Tetracosahexaenoic acid | 0.0246 | 1.2319 | 3267565.0 | 2277387.0 | 364186.0 | ↓ |
| PE(P-16:0/22:6) | 0.0156 | 5.9433 | 2896700.0 | 14423880.0 | 3077155.0 | ↑ |
| PC(P-14:0/20:1) | 0.0103 | 1.7937 | 10699280.0 | 4910509.0 | 2100016.0 | ↓ |
| PC(P-14:0/20:0) | 0.0030 | 2.8815 | 28419070.0 | 118131400.0 | 13010200.0 | ↑ |
| PC(22:5/20:5) | 0.0062 | 6.1496 | 930513.0 | 6254959.0 | 1347488.0 | ↑ |
| PC(20:4/15:0) | 0.0051 | 4.4469 | 796114.0 | 8465376.0 | 2192915.0 | ↑ |
| PC(20:4/20:5) | 0.0495 | 3.5094 | 130473.0 | 4473827.0 | 2822762.0 | ↑ |
| PC(20:2/20:5) | 0.0155 | 4.2808 | 308872.0 | 24230900.0 | 17161150.0 | ↑ |
| PC(20:2/18:1) | 0.0026 | 1.0667 | 28186280.0 | 18558650.0 | 2248608.0 | ↓ |
| PC(18:3/20:4) | 0.0011 | 5.6506 | 337340.0 | 4672639.0 | 1082000.0 | ↑ |
| PC(18:3/20:3) | 0.0240 | 4.3669 | 624374.0 | 57826880.0 | 34357190.0 | ↑ |
| PC(18:2/18:2) | 0.0047 | 3.3760 | 50421790.0 | 14783250.0 | 10299190.0 | ↓ |
| PC(18:1/18:0) | 0.0311 | 2.1327 | 21281040.0 | 8082373.0 | 5397282.0 | ↓ |
| PC(16:0/22:4) | 0.0003 | 1.7680 | 13621020.0 | 6461538.0 | 1448729.0 | ↓ |
| PC(16:0/18:3) | 0.0011 | 3.1548 | 5850577.0 | 1311635.0 | 1208778.0 | ↓ |
| PC(16:0/18:1(9Z)) | 0.0005 | 2.9399 | 278304100.0 | 60010690.0 | 42284630.0 | ↓ |
| PC(14:0/20:0) | 0.0004 | 2.8972 | 36440070.0 | 11360820.0 | 5720418.0 | ↓ |
| Palmitoyl sphingomyelin | 0.0068 | 4.4140 | 52994.6 | 2122648.0 | 859636.0 | ↑ |
| LysoPS 22:6 | 0.0054 | 1.0840 | 18397460.0 | 9087279.0 | 2993908.0 | ↓ |
| LysoPI 20:4 | 0.0040 | 2.1074 | 24764710.0 | 11966930.0 | 3542345.0 | ↓ |
| LysoPI 16:0 | 0.0067 | 3.9675 | 1579116.0 | 7445365.0 | 974664.0 | ↑ |
| LysoPG 18:2 | 0.0001 | 2.4498 | 49670.9 | 252325.0 | 42167.4 | ↑ |
| LysoPC 16:0 | 0.0318 | 1.1088 | 9233437.0 | 5622412.0 | 1562869.0 | ↓ |
| L-Iditol | 0.0466 | 1.5976 | 111715.0 | 76171.8 | 17511.5 | ↓ |
| Indoxyl sulfate | 0.0100 | 1.2887 | 428086.0 | 164850.0 | 109203.0 | ↓ |
| Hypotaurine | 0.0063 | 1.2665 | 20622470.0 | 31243200.0 | 2508228.0 | ↑ |
| Dodecylbenzenesulfonic acid | 0.0402 | 1.9037 | 27294560.0 | 11103280.0 | 9925545.0 | ↓ |
| Docosapentaenoic acid | 0.0096 | 1.4503 | 13478860.0 | 9228259.0 | 1256861.0 | ↓ |
| Docosahexaenoic acid | 0.0057 | 1.2323 | 489317000.0 | 358137500.0 | 38413770.0 | ↓ |
| Deoxycholic acid | 0.0061 | 1.8262 | 380511.0 | 155470.0 | 70954.7 | ↓ |
| Adenine | 0.0152 | 1.1328 | 186837.0 | 124683.0 | 20261.9 | ↓ |
| 3-Hydroxy-3-methylglutaric acid | 0.0304 | 1.1862 | 4546216.0 | 2899978.0 | 696015.0 | ↓ |
| 2-Methylpiperidine | 0.0054 | 1.6248 | 178973.0 | 375353.0 | 62284.2 | ↑ |
| PE(18:0/22:6) | 0.0178 | 3.9980 | 523435.0 | 10757700.0 | 3231775.0 | ↑ |
| 2,3-Methylenesuccinic acid | 0.0413 | 1.9661 | 1233413.0 | 694175.0 | 174538.0 | ↓ |
| PC(18:0/18:2w6) | 0.0065 | 1.8121 | 39532660.0 | 14800590.0 | 7721384.0 | ↓ |
| PC(18:0/22:6) | 0.0002 | 1.4124 | 4998087.0 | 2614750.0 | 448745.0 | ↓ |
| PC(16:0/18:2w6) | 0.0000 | 3.6222 | 607953.0 | 3706539.0 | 655608.0 | ↑ |
| 1-Methylhypoxanthine | 0.0384 | 1.7285 | 6600056.0 | 10285120.0 | 1045852.0 | ↑ |
| 1-Butylimidazole | 0.0149 | 2.4605 | 33230160.0 | 15443910.0 | 5560355.0 | ↓ |
| PC(18:1/18:1) | 0.0002 | 2.8241 | 43071250.0 | 15028060.0 | 5565243.0 | ↓ |
| PC(16:0/16:0) | 0.0002 | 2.8983 | 32616560.0 | 5945123.0 | 5617553.0 | ↓ |
| PC(22:6/22:6) | 0.0016 | 3.9384 | 983602.0 | 6612034.0 | 1076221.0 | ↑ |
| 1,2-Dichloroethane | 0.0067 | 2.2324 | 2019427.0 | 6605116.0 | 1326176.0 | ↑ |
| PC(P-18:0/18:1(9Z)) | 0.0002 | 3.6281 | 5629467.0 | 26390010.0 | 3410378.0 | ↑ |

**Table (d). Differentially PFC Metabolites Between DSS + CUR and DSS Group**

| **MS2Metabolite** | **p value** | **VIP** | **DSS Mean** | **DSS+CUR Mean** | **SE of difference** | **Trends after CUR treatment** |
| --- | --- | --- | --- | --- | --- | --- |
|  |  |  |  |  |  |  |
| Xanthine | 0.0076 | 1.0 | 20012500.0 | 35314170.0 | 4571595.0 | ↑ |
| Tryptophan | 0.0066 | 1.2 | 122185.0 | 255219.0 | 26692.6 | ↑ |
| Triacetin | 0.0242 | 1.6 | 260586.0 | 69585.4 | 64203.9 | ↓ |
| PE(P-16:0/22:6) | 0.0002 | 1.9 | 14423880.0 | 3173347.0 | 2185773.0 | ↓ |
| PC(P-14:0/20:1) | 0.0002 | 3.2 | 5321357.0 | 1100841.0 | 811662.0 | ↓ |
| Phthalic anhydride | 0.0436 | 1.5 | 11108880.0 | 4895008.0 | 2364434.0 | ↓ |
| Phosphocholine | 0.0398 | 3.5 | 117646.0 | 8897868.0 | 2975137.0 | ↑ |
| PE(P-16:0e/0:0) | 0.0170 | 1.5 | 4177053.0 | 2197685.0 | 514242.0 | ↓ |
| PC(22:2/15:0) | 0.0084 | 1.2 | 24575830.0 | 10112150.0 | 2674885.0 | ↓ |
| PC(20:4/20:5) | 0.0169 | 4.0 | 4473827.0 | 17036630.0 | 3210348.0 | ↑ |
| PC(20:2/20:5) | 0.0076 | 3.8 | 4331860.0 | 23027520.0 | 6120328.0 | ↑ |
| PC(20:2/18:1(11Z)) | 0.0003 | 2.5 | 18558650.0 | 5999395.0 | 1736458.0 | ↓ |
| PC(20:1(11Z)/15:0) | 0.0065 | 1.5 | 29918780.0 | 9668984.0 | 5746006.0 | ↓ |
| PC(18:3/20:4) | 0.0014 | 1.8 | 4672639.0 | 16270200.0 | 2147116.0 | ↑ |
| PC(18:3/20:3) | 0.0140 | 4.8 | 2565084.0 | 20533060.0 | 4128640.0 | ↑ |
| PC(18:2/18:2) | 0.0007 | 6.4 | 7627906.0 | 174508300.0 | 42321540.0 | ↑ |
| PC(18:1(9Z)/18:0) | 0.0248 | 1.5 | 48193260.0 | 87379260.0 | 14718700.0 | ↑ |
| PC(18:0/20:5) | 0.0009 | 4.1 | 12424000.0 | 81983980.0 | 17323850.0 | ↑ |
| PC(18:0/20:2) | 0.0185 | 1.4 | 6618422.0 | 12669570.0 | 2301142.0 | ↑ |
| PC(16:0/22:6) | 0.0081 | 5.0 | 40758970.0 | 398455400.0 | 79998750.0 | ↑ |
| PC(16:0/22:4) | 0.0066 | 1.8 | 2982341.0 | 8535237.0 | 2015527.0 | ↑ |
| PC(16:0/18:1(9Z)) | 0.0027 | 2.4 | 23589840.0 | 4491734.0 | 5195975.0 | ↓ |
| Palmitoyl sphingomyelin | 0.0374 | 2.4 | 2122648.0 | 9085112.0 | 2417050.0 | ↑ |
| Palmitamide | 0.0197 | 1.4 | 5271403.0 | 8754759.0 | 1229753.0 | ↑ |
| N-lactoyl-Valine | 0.0417 | 2.5 | 217676.0 | 885609.0 | 373371.0 | ↑ |
| Niacinamide | 0.0175 | 3.1 | 1014124.0 | 187751.0 | 283986.0 | ↓ |
| N,N-Dimethylaniline | 0.0485 | 1.2 | 29857910.0 | 16981220.0 | 4838538.0 | ↓ |
| Maleic acid | 0.0013 | 1.0 | 41234580.0 | 74321230.0 | 6685149.0 | ↑ |
| LysoPS 22:6 | 0.0089 | 1.1 | 9087279.0 | 4335406.0 | 1343224.0 | ↓ |
| LysoPI 20:4 | 0.0313 | 1.6 | 5188639.0 | 2209974.0 | 1319098.0 | ↓ |
| LysoPG 18:2 | 0.0003 | 1.8 | 252325.0 | 1061971.0 | 238152.0 | ↑ |
| LysoPE 18:1 | 0.0102 | 1.8 | 32411320.0 | 17426550.0 | 4048404.0 | ↓ |
| LysoPE 16:0 | 0.0383 | 1.8 | 24009690.0 | 11464340.0 | 2899520.0 | ↓ |
| LysoPC 22:5 | 0.0096 | 1.1 | 712745.0 | 1472453.0 | 252713.0 | ↑ |
| LysoPC 20:1 | 0.0014 | 1.6 | 1962460.0 | 6595998.0 | 1376863.0 | ↑ |
| L-Cysteine-glutathione disulfide | 0.0000 | 1.1 | 2379607.0 | 4840360.0 | 256552.0 | ↑ |
| L-5-Oxoproline | 0.0006 | 1.0 | 126330.0 | 65806.2 | 12988.2 | ↓ |
| Hypotaurine | 0.0002 | 2.0 | 31243200.0 | 74595730.0 | 7332109.0 | ↑ |
| Hydroquinone | 0.0308 | 2.6 | 19249870.0 | 4859869.0 | 5324351.0 | ↓ |
| Glutathione, oxidized | 0.0459 | 1.3 | 7301285.0 | 14028370.0 | 2605066.0 | ↑ |
| Docosapentaenoic acid | 0.0190 | 1.0 | 9228259.0 | 6640957.0 | 864448.0 | ↓ |
| Citric acid | 0.0000 | 1.7 | 94694700.0 | 232827400.0 | 15845850.0 | ↑ |
| Choline | 0.0060 | 2.3 | 41326500.0 | 8033124.0 | 7502976.0 | ↓ |
| Adenine | 0.0008 | 1.5 | 11204090.0 | 5672015.0 | 1179847.0 | ↓ |
| 5,6,7,8-Tetrahydro-4-methylquinoline | 0.0050 | 1.2 | 6145386.0 | 4361295.0 | 367906.0 | ↓ |
| 3-Hydroxyoleylcarnitine | 0.0088 | 1.7 | 3985959.0 | 6968389.0 | 915984.0 | ↑ |
| 3-Furancarboxylic acid | 0.0000 | 2.0 | 13477190.0 | 38772770.0 | 3149413.0 | ↑ |
| 2-Methylpiperidine | 0.0001 | 2.4 | 375353.0 | 1234909.0 | 156524.0 | ↑ |
| 2,2'-Methylene-bis(6-tert-butyl-4 methylphenol) | 0.0072 | 2.3 | 1962557.0 | 952968.0 | 214074.0 | ↓ |
| PC(18:0/18:2w6) | 0.0001 | 2.9 | 14800590.0 | 2022831.0 | 3015978.0 | ↓ |
| PC(18:0/22:6) | 0.0014 | 1.0 | 2614750.0 | 4667643.0 | 478090.0 | ↑ |
| PC(16:0/14:0) | 0.0095 | 4.5 | 7606856.0 | 42930720.0 | 21677840.0 | ↑ |
| PC(16:0/18:2) | 0.0166 | 3.8 | 922293.0 | 20281850.0 | 8096683.0 | ↑ |
| 1-Butylimidazole | 0.0219 | 1.9 | 15443910.0 | 30182050.0 | 4756035.0 | ↑ |
| [PC(18:1/18:1)](https://www.ncbi.nlm.nih.gov/pcsubstance/?term=) | 0.0015 | 2.5 | 5621557.0 | 988303.0 | 1426075.0 | ↓ |
| (-)-Riboflavin | 0.0045 | 1.5 | 936962.0 | 1549498.0 | 165620.0 | ↑ |

**Table (e). Differentially Serum Metabolites Between DSS and Control Group**

|  |  |  |  |  |  |  |
| --- | --- | --- | --- | --- | --- | --- |
| **MS2Metabolite** | **p value** | **VIP** | **DSS Mean** | **Control Mean** | **SEM of difference** | **Trends after DSS treatment** |
| Xanthine | 0.001 | 3.525 | 4388213 | 22723340 | 3468976 | ↓ |
| Taurine | 0.003 | 1.007 | 10077610 | 16801050 | 1765528 | ↓ |
| Pyroglutamylvaline | 0.0001 | 1.94 | 985382 | 516746 | 78802.4 | ↑ |
| Proline | 0.042 | 1.993 | 19250470 | 12347440 | 2790549 | ↑ |
| Plasmenyl-PC 17:0 | 0.036 | 1.111 | 6131558 | 9742665 | 1208992 | ↓ |
| Phenol sulphate | 0.009 | 1.323 | 65741060 | 163547600 | 29551260 | ↓ |
| Niacinamide | 0.003 | 1.131 | 264732 | 405938 | 34131.8 | ↓ |
| Maleic acid | 0.004 | 1.286 | 243422 | 359504 | 34638.9 | ↓ |
| LysoPE 20:3 | 0.046 | 1.055 | 11869510 | 22295280 | 4987873 | ↓ |
| LysoPC 22:5 | 0.0001 | 1.072 | 37416080 | 76232160 | 4761289 | ↓ |
| LysoPC 20:3 | 0.006 | 1.207 | 6066943 | 12261050 | 1199888 | ↓ |
| LysoPC 16:1 | 0.001 | 1.91 | 25252770 | 65659170 | 9144948 | ↓ |
| L-.gamma.-Glutamyl-L-glutamic acid | 0.018 | 1.639 | 107724 | 197332 | 23719.1 | ↓ |
| Inosine | 0.005 | 2.505 | 2799700 | 9428959 | 2207746 | ↓ |
| Hypoxanthine | 0.002 | 1.421 | 879886 | 2160282 | 329251 | ↓ |
| Hydroxyphenyllactic acid | 0.009 | 1.111 | 6726998 | 9374234 | 772387 | ↓ |
| Guanosine 5'-monophosphate | 0.025 | 1.016 | 368519 | 232961 | 41142 | ↑ |
| Guanosine | 0.001 | 1.816 | 276848 | 798835 | 103765 | ↓ |
| Glutathione, oxidized | 0.014 | 1.845 | 17771.7 | 72240.1 | 26403.8 | ↓ |
| Ethyl sulfate | 0.015 | 2.271 | 5924885 | 2572152 | 1220510 | ↑ |
| ent-15-Oxo-16-kauren-19-oic acid | 0.049 | 1.493 | 403339 | 252112 | 69880.9 | ↑ |
| Enoxacin | 0.008 | 1.352 | 16850.2 | 8739.25 | 1807.22 | ↑ |
| Dulcitol | 0.006 | 1.498 | 504675 | 791100 | 86984.1 | ↓ |
| D-Ribose 1-phosphate | 0.008 | 2.198 | 131653 | 298776 | 38562.7 | ↓ |
| Dexpanthenol | 0.033 | 2.819 | 160074 | 820056 | 232891 | ↓ |
| Citric acid | 0.009 | 1.349 | 1791202 | 2626025 | 254037 | ↓ |
| Beclomethasone | 0.005 | 2.356 | 84949.4 | 164657 | 18089.4 | ↓ |
| AMP | 0.001 | 3.279 | 2118497 | 410675 | 417494 | ↑ |
| Adenosine monophosphate | 0.003 | 3.558 | 607478 | 71304.1 | 142108 | ↑ |
| Acylcarnitine 22:6 | 0.04 | 1.032 | 960640 | 1380589 | 180446 | ↓ |
| Acylcarnitine 14:0 | 0.002 | 1.822 | 27799580 | 53822720 | 6133313 | ↓ |
| 3-Hydroxydodecanoic acid | 0.04 | 1.924 | 1446774 | 3300143 | 1034737 | ↓ |
| 2,4-Dihydroxyacetophenone 5-sulfate | 0.0001 | 1.229 | 3822796 | 9749730 | 1203078 | ↓ |
| 1-Mercapto-2-propanone | 0.037 | 1.598 | 2315721 | 3666560 | 579191 | ↓ |

**Table (f). Differentially Serum Metabolites Between DSS + CUR and DSS Group**

| **MS2Metabolite** | **p value** | **VIP** | **DSS+CUR Mean** | **DSS Mean** | **SEM of difference** | **Trends after CUR treatment** |
| --- | --- | --- | --- | --- | --- | --- |
| Uridine 5'-monophosphate | 0.004 | 1.255 | 112256 | 54034.4 | 15414.6 | ↑ |
| Threonic acid | 0.031 | 1.173 | 4203182 | 2574083 | 529650 | ↑ |
| Proline | 0.028 | 1.563 | 13732790 | 19250470 | 2389317 | ↓ |
| Phosphocholine | 0.027 | 2.801 | 12976840 | 40299280 | 9105940 | ↓ |
| Phenol sulphate | 0.020 | 1.731 | 23281950 | 65741060 | 18704010 | ↓ |
| O,O-Diethyl phosphate | 0.021 | 1.390 | 5835161 | 3726024 | 789135 | ↑ |
| N-Hexadecanoylpyrrolidine | 0.013 | 3.047 | 32036950 | 6448647 | 9556907 | ↑ |
| N,N-Dimethylaniline | 0.023 | 1.520 | 9307866 | 16169580 | 3114098 | ↓ |
| Methoxyeugenol | 0.038 | 2.491 | 568560 | 1022828 | 166035 | ↓ |
| Mesaconic acid | 0.038 | 1.673 | 5550121 | 3357406 | 832316 | ↑ |
| LysoPS 22:6 | 0.021 | 1.613 | 124790 | 269227 | 42903.9 | ↓ |
| LysoPG 18:1 | 0.009 | 1.624 | 1982758 | 3614479 | 559335 | ↓ |
| LysoPE 22:4 | 0.028 | 2.684 | 12657670 | 6604753 | 1891019 | ↑ |
| LysoPC 16:1 | 0.048 | 1.006 | 37514310 | 25252770 | 4708127 | ↑ |
| L-Propionylcarnitine | 0.037 | 1.279 | 1893690 | 3070550 | 429824 | ↓ |
| Inosine-5'-monophosphate | 0.016 | 1.974 | 46377.3 | 18332.8 | 5800.24 | ↑ |
| Inosine | 0.012 | 1.542 | 1623138 | 2799700 | 328910 | ↓ |
| Hydroquinone | 0.016 | 2.928 | 50997060 | 16690920 | 12244280 | ↑ |
| Guanosine | 0.040 | 1.153 | 142902 | 276848 | 59825.5 | ↓ |
| Dodecylbenzenesulfonic acid | 0.046 | 2.992 | 1674373 | 4003160 | 952463 | ↓ |
| Dexpanthenol | 0.011 | 2.469 | 490451 | 160074 | 122648 | ↑ |
| D-erythro-Sphinganine-1-phosphate | 0.044 | 1.100 | 7663079 | 4419788 | 1408629 | ↑ |
| AMP | 0.041 | 1.893 | 4541292 | 2118497 | 800703 | ↑ |
| Adenosine monophosphate | 0.035 | 1.427 | 1420010 | 607478 | 329421 | ↑ |
| 3-Hydroxydodecanoic acid | 0.044 | 1.895 | 2656692 | 1446774 | 805294 | ↑ |
| 3-Furancarboxylic acid | 0.009 | 3.059 | 2089428 | 571506 | 494741 | ↑ |
| 3-ethylphenyl Sulfate | 0.017 | 1.918 | 37177430 | 125681900 | 26912800 | ↓ |
| 2-Pyridylacetic acid | 0.013 | 4.731 | 929773 | 330872 | 302078 | ↑ |
| 2,6-Dihydroxybenzoic acid | 0.021 | 1.359 | 2761620 | 4635724 | 662190 | ↓ |
| 1-Mercapto-2-propanone | 0.041 | 1.012 | 747650200 | 945372700 | 83913920 | ↓ |
|  |  |  |  |  |  |  |
|  | | | | | | |

**Table (g). Differentially PFC Lipid Metabolites Between FMT (DSS) and FMT (DSS + CUR) Group**

| **MS2Metabolite** | **p value** | **VIP** | **FMT(DSS) Mean** | **FMT(DSS+CUR) Mean** | **SEM of difference** | **Trends after FMT** |
| --- | --- | --- | --- | --- | --- | --- |
| Xanthine | 0.018 | 1.2 | 4730962.0 | 7507472.0 | 968983.0 | ↑ |
| Vulgarone A | 0.003 | 1.5 | 766580.0 | 1280906.0 | 131904.0 | ↑ |
| Uracil | 0.026 | 1.1 | 11338580.0 | 6186421.0 | 2274083.0 | ↓ |
| Triethyl phosphate | 0.018 | 1.4 | 21203770.0 | 8468149.0 | 5586401.0 | ↓ |
| S-Adenosyl-L-homocysteine | 0.001 | 1.7 | 1097976.0 | 2482557.0 | 351141.0 | ↑ |
| Propionic acid | 0.016 | 1.4 | 2892902.0 | 1621774.0 | 549339.0 | ↓ |
| Proline | 0.048 | 2.1 | 1836212.0 | 336363.0 | 659947.0 | ↓ |
| Plasmenyl-PC 34:1 | 0.001 | 2.1 | 9657488.0 | 2515075.0 | 1745326.0 | ↓ |
| Plasmenyl-PC 34:0 | 0.033 | 1.3 | 11405190.0 | 29032380.0 | 8747812.0 | ↑ |
| Phosphocholine | 0.019 | 1.2 | 7043786.0 | 12664000.0 | 2012086.0 | ↑ |
| PE(P-16:0e/0:0) | 0.003 | 1.8 | 7915738.0 | 3268081.0 | 1508042.0 | ↓ |
| p-Cresol sulfate | 0.004 | 2.6 | 78654.7 | 2900825.0 | 2195779.0 | ↑ |
| PC(22:5/20:5) | 0.011 | 3.0 | 10218960.0 | 33806050.0 | 10921660.0 | ↑ |
| PC(22:2/15:0) | 0.029 | 1.3 | 17818410.0 | 11179860.0 | 2614840.0 | ↓ |
| PC(20:4/15:0) | 0.001 | 2.9 | 1578870.0 | 7533529.0 | 1249715.0 | ↑ |
| PC(20:4/20:5() | 0.001 | 4.4 | 177195.0 | 14243170.0 | 1879658.0 | ↑ |
| PC(20:2/20:5) | 0.001 | 3.5 | 813206.0 | 21584720.0 | 3328853.0 | ↑ |
| PC(20:2/18:1(11Z)) | 0.001 | 1.8 | 38221960.0 | 13680680.0 | 6816574.0 | ↓ |
| PC(20:1(11Z)/15:0) | 0.022 | 1.4 | 35865440.0 | 18871450.0 | 6321050.0 | ↓ |
| PC(18:3/20:4) | 0.008 | 2.0 | 4473263.0 | 10138160.0 | 2309305.0 | ↑ |
| PC(18:3/20:3) | 0.000 | 4.8 | 824547.0 | 167562100.0 | 41453260.0 | ↑ |
| PC(18:2/18:2) | 0.001 | 2.9 | 13626340.0 | 221866200.0 | 52493280.0 | ↑ |
| PC(18:1(9Z)/18:0) | 0.010 | 1.4 | 116236800.0 | 247727200.0 | 44797420.0 | ↑ |
| PC(18:0/20:5) | 0.009 | 1.8 | 15833300.0 | 56632640.0 | 14796740.0 | ↑ |
| PC(18:0/20:2) | 0.015 | 1.5 | 3383818.0 | 12012170.0 | 4781925.0 | ↑ |
| PC(16:0/22:6) | 0.001 | 4.6 | 2684910.0 | 346861000.0 | 73584820.0 | ↑ |
| PC(16:0/18:1(9Z)) | 0.002 | 2.7 | 460226200.0 | 100344200.0 | 113926400 | ↓ |
| PC(14:0/20:0) | 0.038 | 1.0 | 30996060.0 | 15275330.0 | 6539166.0 | ↓ |
| Morpholine | 0.017 | 1.0 | 897791.0 | 1361673.0 | 177013.0 | ↑ |
| Methylphosphate | 0.001 | 1.4 | 28797790.0 | 50229000.0 | 3931068.0 | ↑ |
| LysoPS 22:6 | 0.001 | 1.6 | 17984040.0 | 7723432.0 | 1971940.0 | ↓ |
| LysoPI 20:4 | 0.002 | 2.2 | 15927910.0 | 4299370.0 | 3549621.0 | ↓ |
| LysoPI 18:2 | 0.003 | 1.4 | 92695.7 | 44120.4 | 12152.8 | ↓ |
| LysoPI 16:0 | 0.001 | 2.8 | 2680628.0 | 28463000.0 | 6404201.0 | ↑ |
| LysoPG 18:2 | 0.001 | 2.8 | 131910.0 | 1174094.0 | 220891.0 | ↑ |
| LysoPE 18:2 | 0.005 | 1.4 | 1235094.0 | 2257717.0 | 282954.0 | ↑ |
| LysoPE 16:0 | 0.002 | 1.2 | 446860.0 | 694023.0 | 50602.8 | ↑ |
| LysoPC 20:3 | 0.001 | 1.4 | 189133.0 | 323320.0 | 22066.0 | ↑ |
| LysoPC 18:2 | 0.014 | 1.1 | 134132.0 | 92776.5 | 12884.2 | ↓ |
| L-(-)-Phenylalanine | 0.004 | 1.3 | 463745.0 | 290655.0 | 60739.5 | ↓ |
| Hypoxanthine | 0.021 | 2.4 | 23887510.0 | 4531798.0 | 5512098.0 | ↓ |
| Hypotaurine | 0.001 | 1.3 | 255341.0 | 159909.0 | 13848.5 | ↓ |
| Glycerol tripropanoate | 0.005 | 1.0 | 32355860.0 | 20612690.0 | 3698730.0 | ↓ |
| Glutathione, oxidized | 0.001 | 2.4 | 3313921.0 | 11605340.0 | 790577.0 | ↑ |
| Dodecylbenzenesulfonic acid | 0.001 | 1.5 | 6327243.0 | 2539277.0 | 1083873.0 | ↓ |
| Docosapentaenoic acid | 0.001 | 1.1 | 14230000.0 | 9041206.0 | 1011635.0 | ↓ |
| Dihydromethysticin | 0.001 | 1.4 | 22306930.0 | 11354920.0 | 2644656.0 | ↓ |
| Dexpanthenol | 0.026 | 1.3 | 149360.0 | 267737.0 | 63038.0 | ↑ |
| D-erythro-Sphingosine-1-phosphate | 0.002 | 2.4 | 293898.0 | 91041.6 | 54081.6 | ↓ |
| Deoxyadenosine | 0.001 | 1.9 | 11189910.0 | 4760329.0 | 912856.0 | ↓ |
| D-(+)-Pantothenic acid | 0.001 | 1.3 | 36334630.0 | 65538560.0 | 7054783.0 | ↑ |
| Citric acid | 0.001 | 1.2 | 190497000.0 | 307951400.0 | 32424020.0 | ↑ |
| Cis-8,11,14,17-Eicosatetraenoic acid | 0.001 | 1.2 | 493974500.0 | 319993300.0 | 38924040.0 | ↓ |
| Beta-Alanine | 0.004 | 1.0 | 111887.0 | 162600.0 | 14211.1 | ↑ |
| all-trans-Retinoic acid | 0.010 | 1.0 | 842042.0 | 1178673.0 | 118644.0 | ↑ |
| Adenosine 5'-diphosphate | 0.003 | 1.2 | 10616690.0 | 17292260.0 | 1972718.0 | ↑ |
| Adenine | 0.050 | 1.1 | 188900.0 | 282376.0 | 36788.2 | ↑ |
| 5-Hydroxy-6E,8Z,11Z,14Z-eicosatetraenoic acid, 1,5-lactone | 0.014 | 1.3 | 2243272.0 | 4127627.0 | 694282.0 | ↑ |
| 5,6,7,8-Tetrahydro-4-methylquinoline | 0.034 | 1.4 | 9599499.0 | 6147330.0 | 1294017.0 | ↓ |
| 3-Hydroxyoleylcarnitine | 0.001 | 1.4 | 3129276.0 | 5501599.0 | 535982.0 | ↑ |
| 3-Hydroxybenzyl alcohol | 0.011 | 1.5 | 1336071.0 | 648819.0 | 304427.0 | ↓ |
| 3-Hydroxy-3-methylglutaric acid | 0.001 | 1.5 | 7445663.0 | 3180118.0 | 1040427.0 | ↓ |
| 2-Nitrophenol | 0.007 | 1.1 | 2573433.0 | 1610540.0 | 359596.0 | ↓ |
| 2-Methylpiperidine | 0.001 | 2.5 | 201844.0 | 1018510.0 | 154761.0 | ↑ |
| 2-Docosahexaenoyl-1-stearoyl-sn-glycero-3-phosphoethanolamine | 0.001 | 3.3 | 2800347.0 | 21032890.0 | 2238833.0 | ↑ |
| 2,6-Dimethylphenol | 0.020 | 1.1 | 58075470.0 | 36410870.0 | 7407486.0 | ↓ |
| 2-(3-Phenylpropyl)tetrahydrofuran | 0.024 | 1.1 | 775034.0 | 1241283.0 | 219622.0 | ↑ |
| 1-Stearoyl-2-linoleoyl-sn-glycero-3-phosphocholine | 0.001 | 2.7 | 24973080.0 | 3051713.0 | 5405824.0 | ↓ |
| 1-Palmitoyl-2-linoleoyl-sn-glycero-3-phosphocholine | 0.001 | 2.4 | 819555.0 | 3588743.0 | 668110.0 | ↑ |
| 1-Methylhydantoin | 0.001 | 1.2 | 743451.0 | 1164242.0 | 89250.8 | ↑ |
| 12S-Hydroxy-5Z,8Z,10E,14Z-eicosatetraenoic acid | 0.015 | 1.7 | 3046303.0 | 6796108.0 | 1692765.0 | ↑ |
| 1,2-dioleoyl-sn-glycero-3-phosphatidylcholine | 0.003 | 2.6 | 384544300.0 | 146521600.0 | 102961500.0 | ↓ |
| 1,2-Dihexadecanoyl-sn-glycero-3-phosphocholine | 0.001 | 1.9 | 4041617.0 | 817612.0 | 1192138.0 | ↓ |
| 1,2-Didocosahexaenoyl-sn-glycero-3-phosphocholine | 0.036 | 1.5 | 3048613.0 | 5235449.0 | 1092216.0 | ↑ |
| 1-(1Z-Octadecenyl)-2-(9Z-octadecenoyl)-sn-glycero-3-phosphocholine | 0.029 | 1.4 | 2226339.0 | 4727843.0 | 1043901.0 | ↑ |
| (S)-2-Azetidinecarboxylic acid | 0.003 | 1.3 | 192745.0 | 103854.0 | 26647.0 | ↓ |
