# Supplementary figures and images for: Curcumin Alleviates DSS-Induced Anxiety-Like Behaviors via the Microbial-Brain-Gut Axis
Source: Oxid Med Cell Longev. 2022 Mar 18;2022:6244757. doi: 10.1155/2022/6244757 (PMC8957039; doi:10.1155/2022/6244757)

A

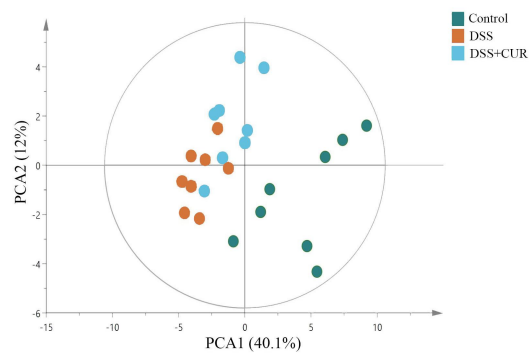

B

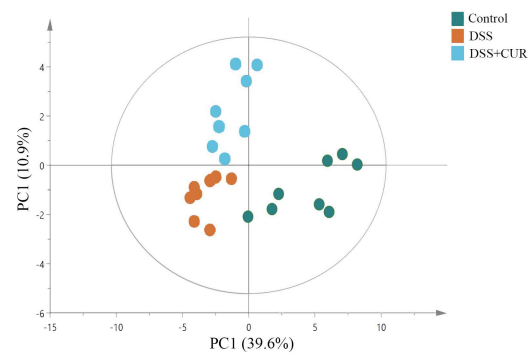

C

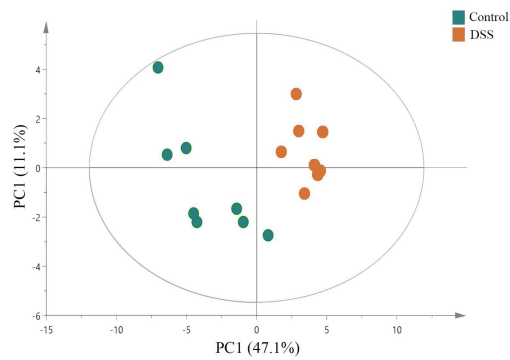

D

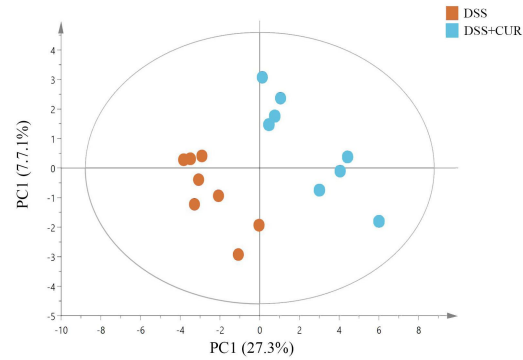

E

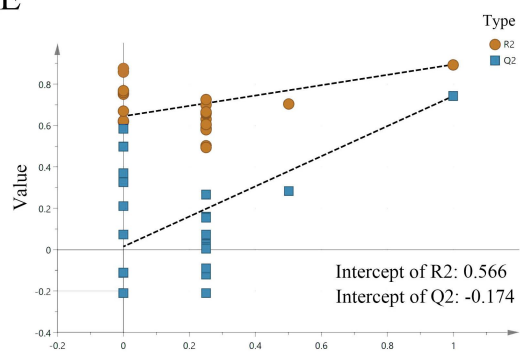

F

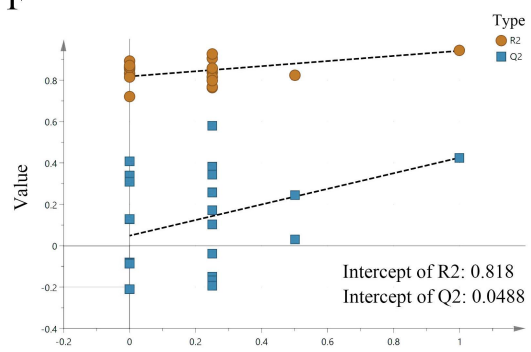

G

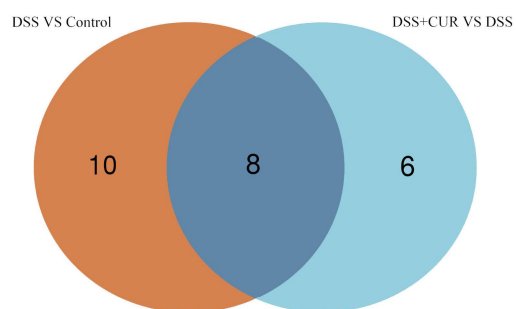

H

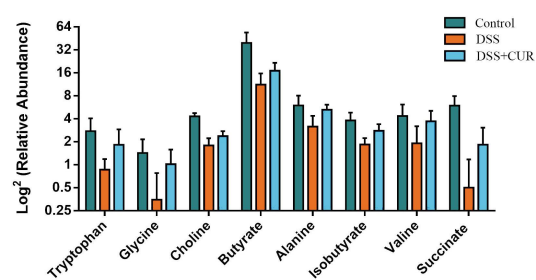

Supplement: Supplementary 2 — Figure supplement 1: fecal metabolic analysis between control, DSS, and DSS + CUR groups (n = 8/group). [file 6244757.f2.pdf]

A

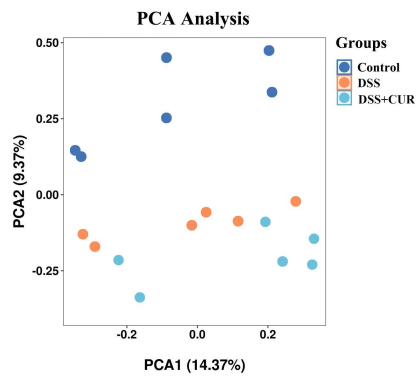

B

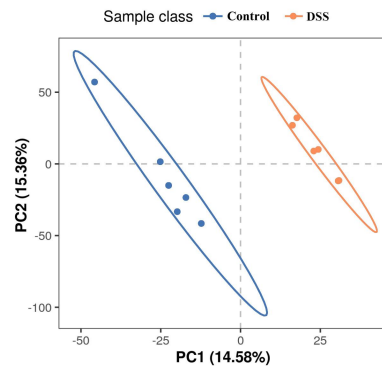

C

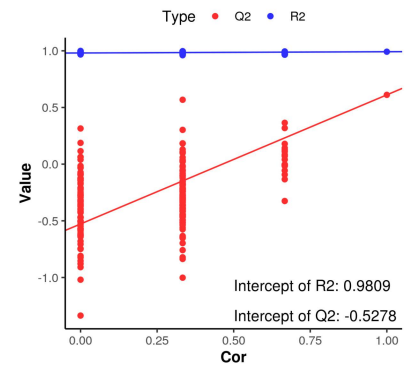

D

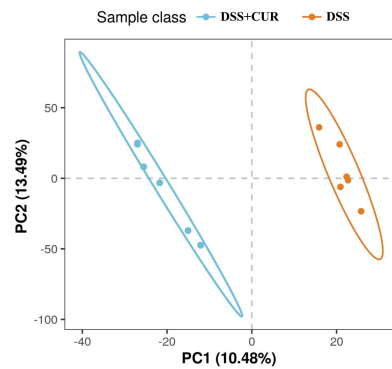

E

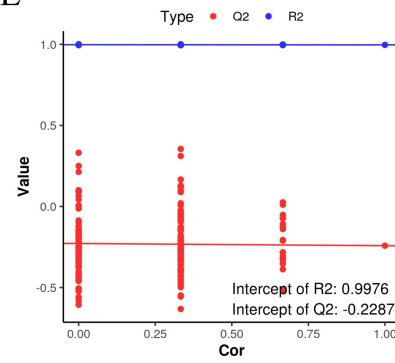

F

### KEGG Enrichment Scatter Plot

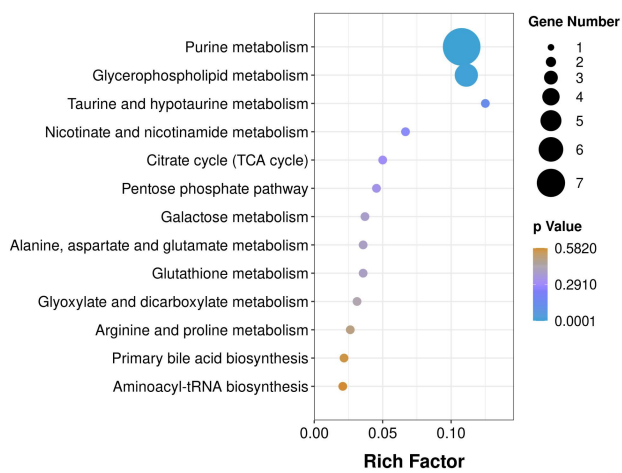

G

### KEGG Enrichment Scatter Plot

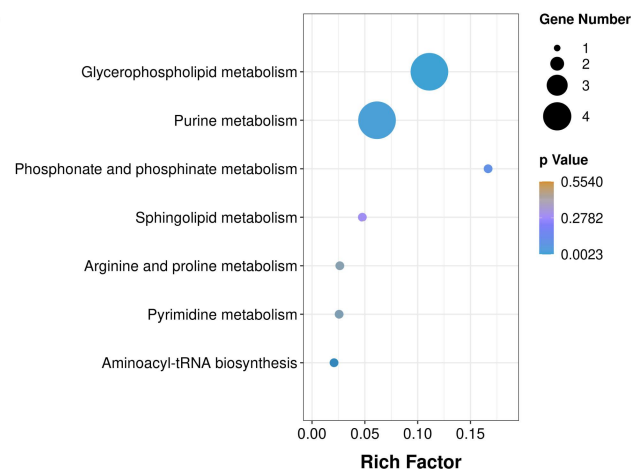

H

### DSS VS Control

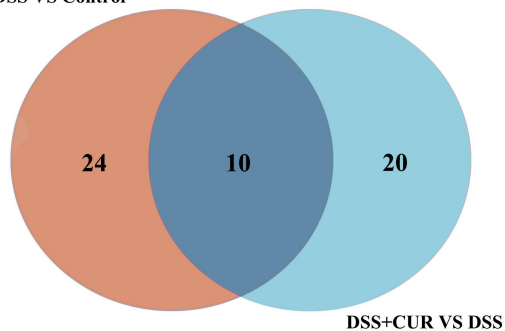

I

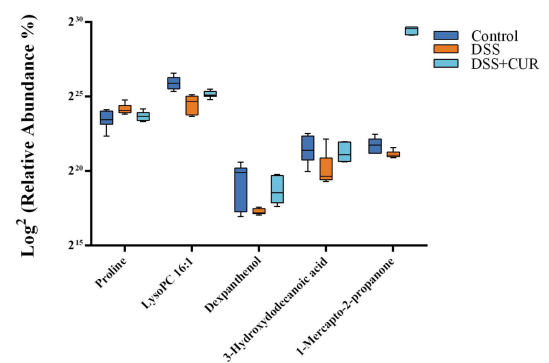

Supplement: Supplementary 3 — Figure supplementary 2: serum metabolic analysis between control, DSS, and DSS + CUR groups (n = 6/group). [file 6244757.f3.pdf]

A

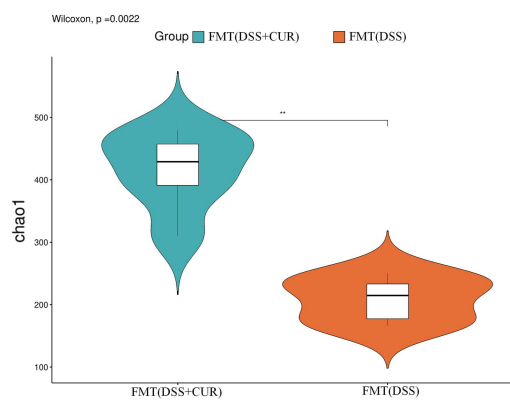

B

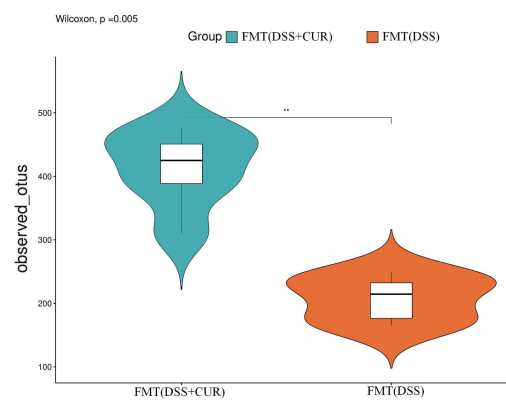

C

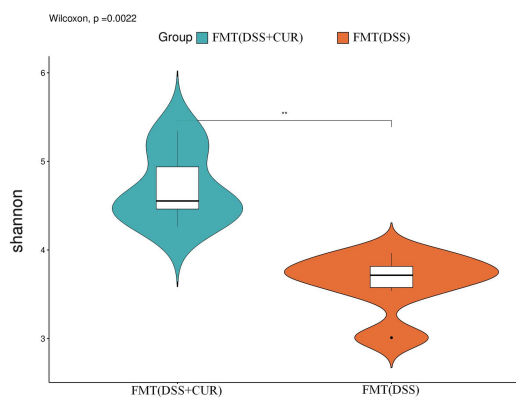

D

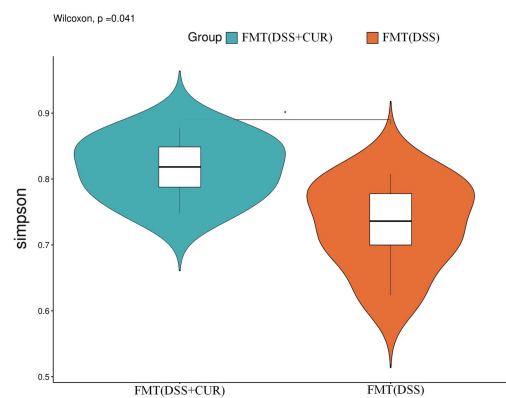

E

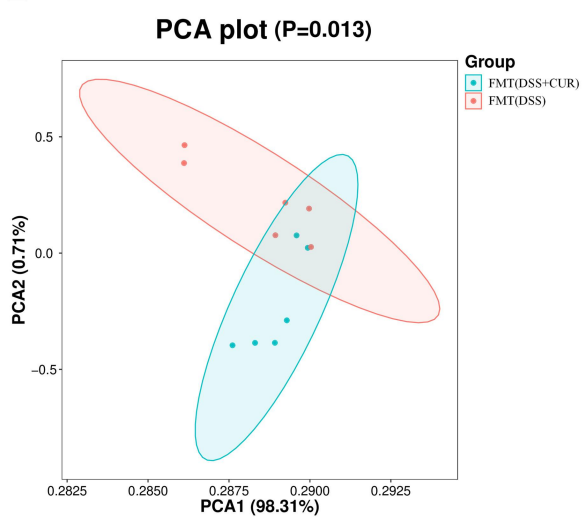

F

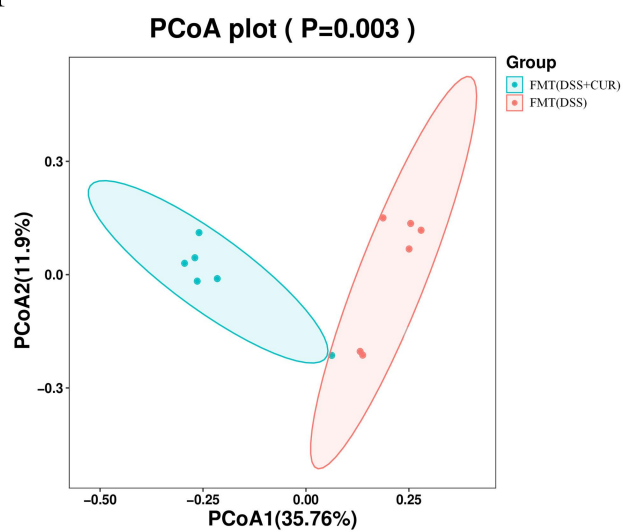

Supplement: Supplementary 4 — Figure supplementary 3: the alpha diversity and beta diversity of gut microbiota between FMT (DSS) group and FMT (DSS + CUR) group (n = 6/group). [file 6244757.f4.pdf]

A

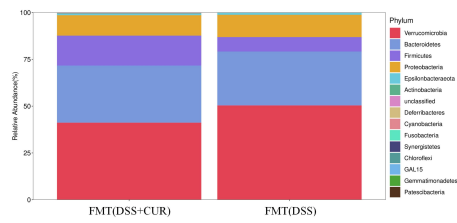

B

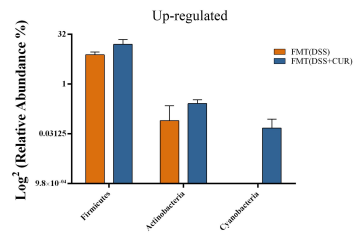

C

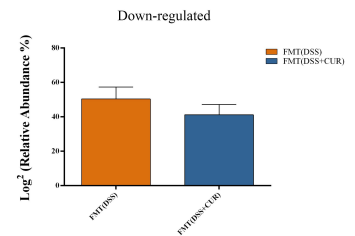

D

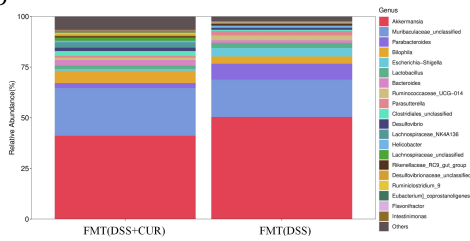

E

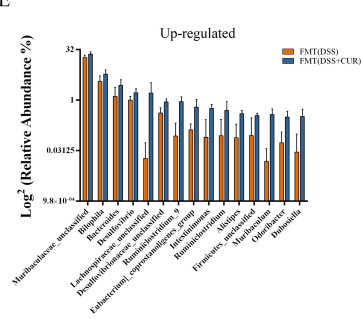

F

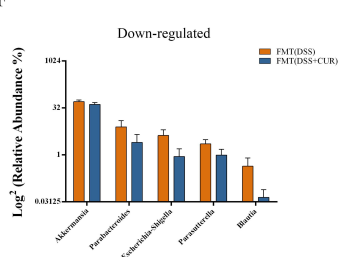

Supplement: Supplementary 5 — Figure supplementary 4: composition of gut microbiota between FMT (DSS) group and FMT (DSS + CUR) group (n = 6/group). [file 6244757.f5.pdf]
